# Gene expression and epigenetics reveal species-specific mechanisms acting upon common molecular pathways in the evolution of task division in bees

**DOI:** 10.1038/s41598-020-75432-8

**Published:** 2021-02-11

**Authors:** Natalia de Souza Araujo, Maria Cristina Arias

**Affiliations:** 1 Department of Genetics and Evolutionary Biology, Universidade de São Paulo, Rua Do Matão, 277, São Paulo, SP 05508-090 Brazil; 2 Present Address: Department of Evolutionary Biology and Ecology, Interuniversity Institute of Bioinformatics in Brussels, Université Libre de Bruxelles, Avenue F.D. Roosevelt, 50, 1050 Brussels, Belgium

**Keywords:** Molecular evolution, Social evolution, Epigenetics, Transcriptomics

## Abstract

A striking feature of advanced insect societies is the existence of workers that forgo reproduction. Two broad types of workers exist in eusocial bees: nurses who care for their young siblings and the queen, and foragers who guard the nest and forage for food. Comparisons between these two worker subcastes have been performed in honeybees, but data from other bees are scarce. To understand whether similar molecular mechanisms are involved in nurse-forager differences across distinct species, we compared gene expression and DNA methylation profiles between nurses and foragers of the buff-tailed bumblebee *Bombus terrestris* and the stingless bee *Tetragonisca angustula.* These datasets were then compared to previous findings from honeybees. Our analyses revealed that although the expression pattern of genes is often species-specific, many of the biological processes and molecular pathways involved are common. Moreover, the correlation between gene expression and DNA methylation was dependent on the nucleotide context, and non-CG methylation appeared to be a relevant factor in the behavioral changes of the workers. In summary, task specialization in worker bees is characterized by a plastic and mosaic molecular pattern, with species-specific mechanisms acting upon broad common pathways across species.

## Introduction

Caste specialization in eusocial insects is a notorious example of polyphenism, where multiple morphological and behavioral phenotypes emerge from the same genotype^1,2^. In social Hymenoptera (bees, wasps and ants), queen and worker castes perform distinct functions in the colony. While queens undertake reproductive duties, workers perform all the other necessary tasks for nest maintenance and growth^3^. Two broad categories of workers exist in eusocial bees: nurses and foragers^4,5^. Nurses are responsible for comb construction, offspring/queen care and internal colony maintenance, while foragers perform tasks related to external colony defense and resource provisioning^5,6^. In advanced eusocial bee species, such as honeybees, worker subcastes are mainly age determined, in which younger bees are nurses, and as they become older, they switch to being foragers^7,8^. In primitively eusocial species^9^, such as the bumblebees, specialization in worker subcastes is not so straightforward, and the same individual may alternate between foraging and nursing many times during its life span ^10,11^.

Many studies have investigated the differences in the worker subcastes of the highly eusocial honeybee (*Apis*). Indeed, gene expression comparisons have identified expression differences between subcastes^1,5,7,12,13^, and have even been used to predict neurogenomic states in individual bees^14^. Similarly, profiles of DNA methylation, an epigenetic mark that likely underpins gene expression differences, were directly correlated with worker tasks^15,16^. Interestingly, studies showed that specific genes are differentially methylated according to the worker subcaste, and foragers that are forced to revert to nursing restore more than half of the nursing-specific DNA methylation marks^17,18^.

It is plausible that many of the molecular differences between honeybee foragers and nurses could have arisen later in the evolution of this lineage. To broadly understand how subcastes evolved, it is necessary to differentiate more recent changes—that could be species-specific—from those that are shared across species and thus likely ancestral. Two alternative, but not mutually exclusive, hypotheses concerning the evolution of sociality focus on the relevance of conserved versus new genes^19,20^. The first is the toolkit hypothesis, which is based on evolutionary developmental biology findings. It predicts that the convergence observed in sociality is built over conserved molecular and physiological networks shared across the different species^3,21^. The second comes from an increasing number of high throughput sequencing studies that advocate for the relevance of taxonomically restricted genes and regulatory pathways in the evolution of behavioral traits^22–28^. Most likely, these two molecular mechanisms are complementary and may have interplayed in the evolution of eusociality, but their proportional contributions to convergent social traits are still debatable^20,29–32^.

Similar to honeybees, the highly eusocial stingless bees have an age-based division of labor^33^; however, their most common ancestor existed 50 to 80 million years ago^34,35^. To date, no global expression or epigenetic studies have been performed in stingless bees to understand worker task specialization. Similarly, while primitively eusocial bumblebees are widely studied as ecological models and represent important wild and managed pollinators, little is known about the molecular underpinning for the differences between its worker subcastes. In large part, studies have been restricted to only a few genes, leaving many open questions^36–38^. A major limiting element for these studies is that these species display a somewhat fluctuating division of labor with indistinctive separation between subcastes^11,36,38^. The characterization of work specialization in bumblebees is essential for comprehending the full spectrum of eusociality, as these bees clearly diverge from highly eusocial species in a number of other traits, including the reduced number of individuals per colony and an annual life cycle^9^.

We aim to fill in this knowledge gap through the analyses of the global gene expression differences between nurses and foragers, and the characterization of DNA methylation profiles in nurses of two eusocial bee species, the primitively eusocial buff-tailed bumblebee, *Bombus terrestris*, and the highly eusocial stingless bee, *Tetragonisca angustula*. Combined, these two bee species and the honeybee represent the three evolutionary branches of eusocial corbiculates that share a common social origin^39^. Hence, in addition to using the generated datasets to uncover unique and more recent transcriptional and epigenetic architectures linked to task division in *B. terrestris* and *T. angustula*, we also included previous *A. mellifera* data in our analyses to verify whether common genes and pathways could be involved in task specialization across all eusocial bee groups.

## Results

### Reference transcriptome assemblies

As a reference for both species, we built a transcriptome of superTranscripts^40^. Briefly, multiple transcripts from the same gene are represented in a single sequence, based on read alignments. Herein, *B. terrestris* workers had 27,987 superTranscripts, of which 431 were potentially long non-coding RNAs (lncRNAs), and 21,638 (77.3%) were annotated. The final *T. angustula* assembly contained 33,065 superTranscripts and was mostly complete. We found that 26,623 superTranscripts (80.5%) had high sequence similarity to known protein-coding genes from other species in the UniRef90 database, and 347 were considered lncRNAs (transcriptomes available at https://github.com/nat2bee/Foragers_vs_Nurses). The ratios of complete hymenopteran BUSCO orthologs found in *B. terrestris* and *T. angustula* transcriptomes were 91.9% and 86.2%, respectively. A summary of major quality parameters from the two species datasets can be found in Supplementary Table SI.

### Differential expression analyses in *Bombus terrestris*

Since task division in *B. terrestris* workers is a plastic behavior^10,36^, we performed a principal component analysis of the normalized read counts as an additional verification step to validate our sampling method. As expected, the main components clustered nurse and forager samples separately (Supplementary Figure S1). We identified 1,203 differentially expressed superTranscripts between the two worker groups (Supplementary Figure S2), whereby 436 superTranscripts were more highly expressed in nurses (Supplementary Information S2), and 767 were more highly expressed in foragers (Supplementary Information S3). The majority of these superTranscripts (77.3% of the nurses biased and 72.6% of the foragers biased) have similarity to known protein-coding genes, while respectively three and one are possible lncRNAs. Moreover, among the differentially expressed superTranscripts, five Gene Ontology (GO) biological process terms (“transposition”, “DNA-mediated, transposition”; “DNA integration”; “DNA recombination”; and “pseudouridine synthesis”) were overrepresented (*p* < 0.01; Supplementary Table SII).

### Differential expression analyses in *Tetragonisca angustula*

In *T. angustula*, a total of 241 superTranscripts were differentially expressed between nurses and foragers (Supplementary Figure S2). Among these, 179 had higher levels of expression in nurses, with 157 genes having a significant blast hit to protein databases (Supplementary Information S4). Foragers had 62 superTranscripts that were more highly expressed than in nurses, of which 59 were annotated (Supplementary Information S5). Subsequent analyses revealed that 30 GO terms for biological process (BP) were enriched in the tested set of differentially expressed superTranscripts when compared to the entire transcriptome (p < 0.01; Supplementary Table SII). Notable examples include processes related to mitochondrial metabolism (“aerobic respiration”; “respiratory electron transport chain”; “oxidative phosphorylation” and “mitochondrial ATP synthesis coupled electron transport”) and other metabolic processes (“lipid metabolic process” and “carbohydrate metabolic process”).

### Taxonomically restricted genes

To identify taxonomically restricted genes, we predicted the open read frames (ORFs) of the assembled superTranscripts and compared the resulting amino acid sequences with the proteomes of eight other Apinae species available at NCBI. Besides our data, we have included in this analysis two species per corbiculate clade (*Apis cerana*, *Apis mellifera*, *Bombus impatiens*, *Bombus terrestris*, *Euglossa dilemma, Eufriesea mexicana*, *Frieseomelitta varia* and *Melipona quadrifasciata*) and one external group (*Habropoda laboriosa*). We used OrthoFinder^41^ to identify orthogroups among all species and classified them according to the species in which they occurred. Overall, OrthoFinder assigned 209,654 genes (91.2% of the total) to 16,602 orthogroups, 6326 of which were present in all of the analyzed species. As expected, the number of unassigned genes were, in general, more substantial in our datasets than in the NCBI proteomes (Supplementary Table SIII). This result is likely due to differences in the filtering and curation processes of our transcriptomes when compared to the NCBI annotations.

In our *B. terrestris* transcriptome, 29,116 (89.8%) of the predicted proteins were placed in orthogroups, and 3312 (10.2%) were unassigned. While for *T. angustula*, 29,408 (78.6%) proteins were placed in orthogroups, and 7988 (21.4%) were unassigned. From the predicted proteins identified as differentially expressed in *B. terrestris*, 86.85% (1,162) were assigned to 962 orthogroups, and 13.15% (176) were unassigned, while in *T. angustula* 88.43% (214) were assigned to 157 orthogroups and 11.57% (28) were unassigned. Only one of the orthogroups differentially expressed in *T. angustula* was from a single-copy ortholog. As the unassigned proteins have no support from other closely related sequences, they may either represent new or incorrectly assembled/annotated genes. Since we included two *B. terrestris* datasets (our transcriptome and the database annotation) and still found a high number of unassigned genes in the transcriptomic data, we decided to consider all the unassigned genes (in *B. terrestris* and *T. angustula*) as probable errors. Consequently, only the genes assigned to orthogroups were considered for the taxonomically restricted gene analyses.

**Figure 1:**
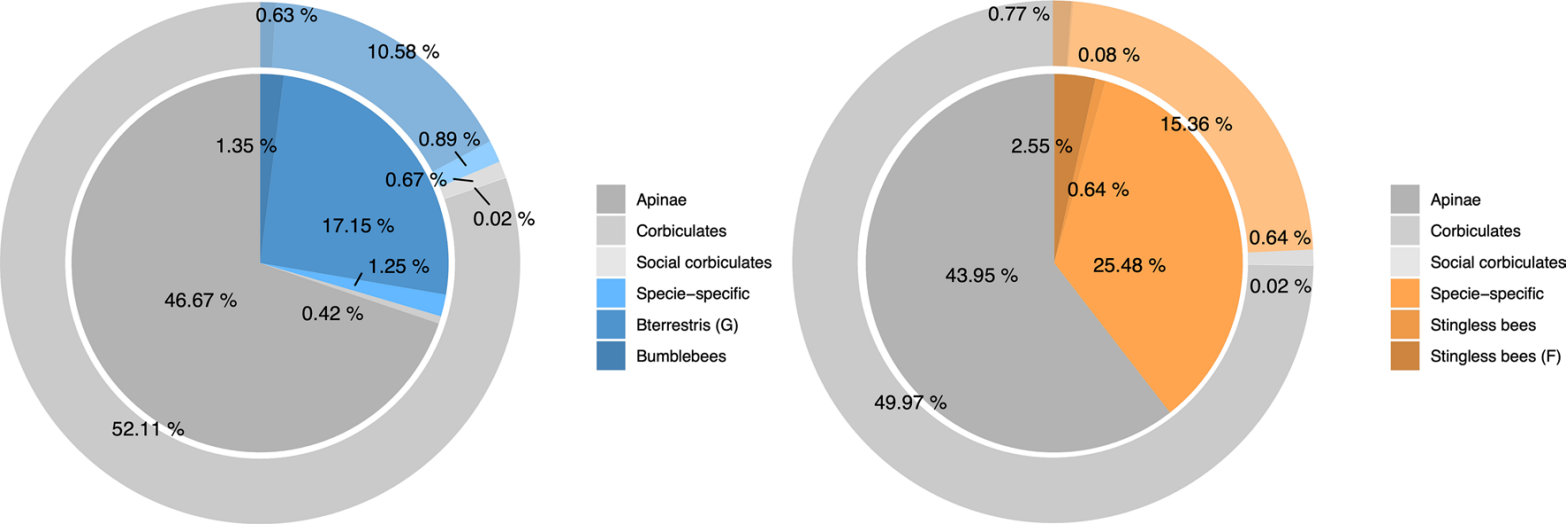
Proportion of conserved and taxonomically restricted orthogroups in *B. terrestris* (left) and *T. angustula* (right) transcriptomes. Inner circles represent the proportion within the differentially expressed genes between nurses and foragers, and outer circles show the proportion in the entire transcriptome. Gray shades represent classes of more taxonomically conserved orthogroups, and shades of blue and orange represent taxonomically restricted classes.

Based on these analyses, we firstly defined three taxonomically conserved classes for the orthogroups: “apinae”, present in all species; “corbiculates” present in all corbiculate lineages; and the “social corbiculates”, only present in honeybees, bumblebees and stingless bees (Supplementary Table SIV). Secondly, other three classes per species defined the taxonomically restricted orthogroups. For *B. terrestris* these classes were: “bumblebees”, orthogroups of the bumblebee clade; “bterrestris (G)”, orthogroups present in our transcriptome that may or may not occur in the *B. terrestris* proteome; and “species-specific”, orthogroups that occur only in the *B. terrestris* datasets (Supplementary Table SIV). The taxonomically restricted classes for *T. angustula* were: “stingless bees (F)”, orthogroups shared by all the stingless bees; “stingless bees”, orthogroups occurring in all stingless bees but ignoring the orthogroups absent in *F. varia*; and the “species-specific” orthogroups that occur only in *T. angustula*. The number and proportion of orthogroups included in each taxonomic category are presented in Supplementary Table SIV and Fig. 1.

Overall, there was an increase in the proportion of taxonomically restricted orthogroups from all three categories among the differentially expressed genes between nurses and foragers when compared to the entire transcriptome (Fig. 1). This difference illustrates the relative importance of new genes in worker specialization, even though genes from more conserved orthogroups still accounted for a large portion of the biased genes.

### DNA methylation in worker genes

**Figure 2:**
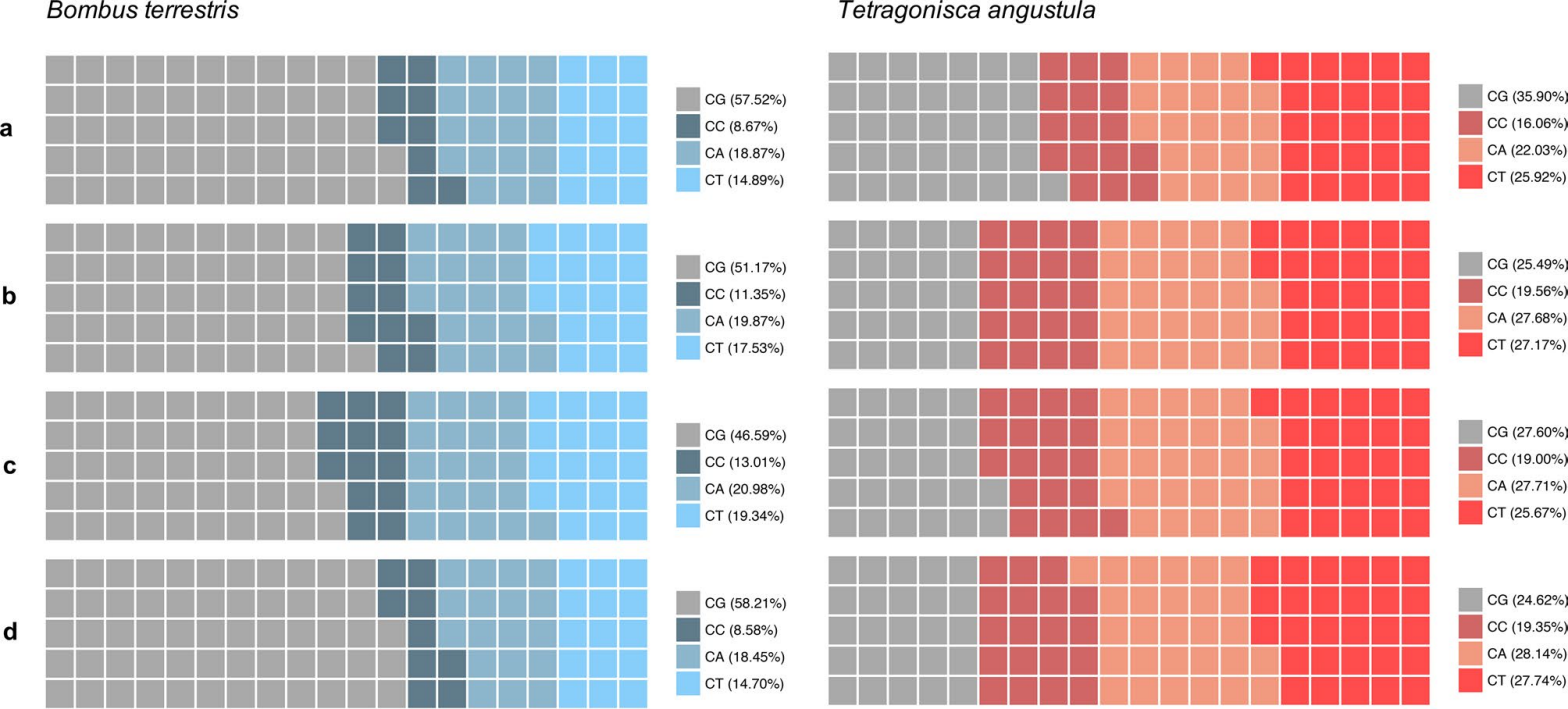
Nucleotide context in which the methylated cytosines occur proportionally to all methylated cytosines reported in nurses of *B. terrestris* and *T. angustula*, in distinct gene sets. **a **in the entire transcriptome; **b** in the differentially expressed superTranscripts between foragers and nurses; **c **in the superTranscripts with higher expression levels in foragers; **d** in the superTranscripts with higher expression levels in nurses. Gray squares represent methylation at the CG context; methylation in non-CG context is illustrated in different shades of blue for *B. terrestris* and shades of red for *T. angustula*. One square ≈ 1%, and considering all of the mC reported sums up to 100%.

**Figure 3:**
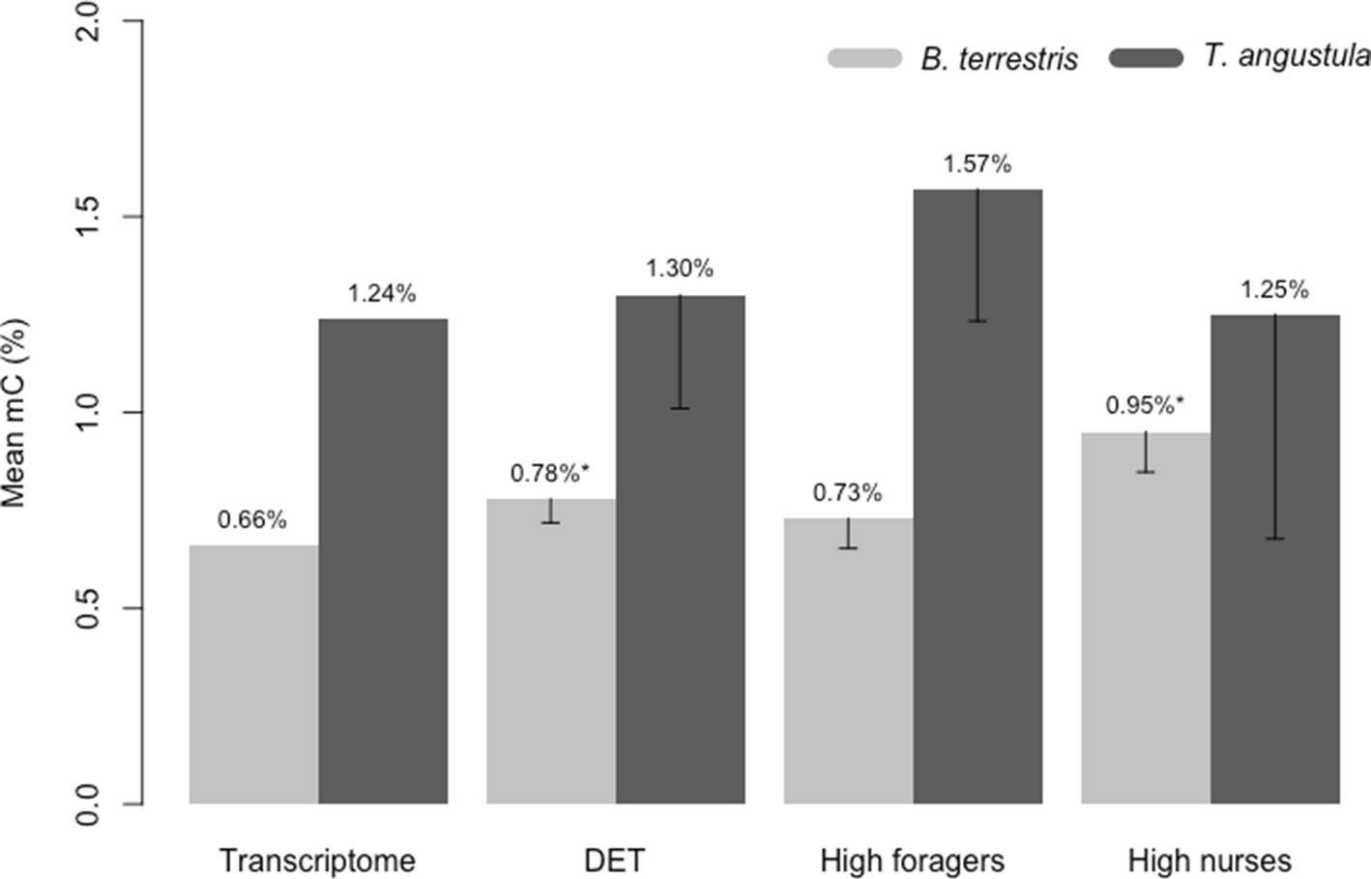
Mean mC levels in distinct gene sets of *B. terrestris* and *T. angustula* nurses. Transcriptome—refers to the values observed in the complete transcriptome; DET—differentially expressed superTranscripts between nurses and foragers; High foragers—superTranscripts with higher expression levels in foragers when compared to nurses; High nurses—superTranscripts with higher expression levels in nurses when compared to foragers. *Significantly different from the global transcriptomic mean, with *p* < 0.01 at 95% CI; confidence interval bars of the statistical tests of significance are shown.

Whole bisulfite sequencing (WBS) from *B. terrestris* and *T. angustula* nurses was used to screen DNA methylation patterns in the entire transcriptome and among the differentially expressed superTranscripts. Since *T. angustula* lacks a reference genome and because most of the DNA methylation reported in bees occurs within gene exons^15^, we performed methylation analyses by mapping bisulfite sequenced reads to the transcriptomes and not the genomes (complete estimations available at https://github.com/nat2bee/Foragers_vs_Nurses). In *B. terrestris*, 23.14% of all cytosine sites are in the CG (cytosine/guanine) context. This proportion is higher than in *T. angustula,* where 15.44% of all C sites available occur in the CG context. This finding could explain the higher proportion of CG methylation observed in the bumblebee (Fig. 2). Nevertheless, in both species, DNA methylation at the CG context was enriched, meaning that there was more DNA methylation at the CG context than it would be expected simply based on the proportion of sites available. Furthermore, global methylation (mC) levels in the superTranscripts were higher in *T. angustula* (mean mC 1.24%) than in *B. terrestris* (mean mC 0.66%) (Fig. 3).

In both species, the differentially expressed superTranscripts had higher levels of methylation than the overall transcriptomic mean (Fig. 3); however, this difference was only significant in *B. terrestris* (*B. terrestris* p = 6.267e−4, *T. angustula* *p* = 0.3669 at 95% CI). While in *B. terrestris*, this increase was mostly due to the greater methylation level of superTranscripts highly expressed in nurses, the mean mC level of the highly expressed superTranscripts in *B. terrestris* nurses was 43.93% higher than the global transcriptomic mean (*p* = 1.339e−06 at 95% CI). In *T. angustula* superTranscripts highly expressed in foragers were the more methylated ones (Fig. 3); however, this result was not at a significant level when compared to the overall mean (*p* = 0.05355 at 95% CI). The nucleotide context in which the methylated cytosines occurred also varied in each gene subset (Fig. 2). There was an overall reduction in the contribution of CG methylation in the subset of differentially expressed superTranscripts when compared to the entire transcriptome, except for superTranscripts highly expressed in *B. terrestris* nurses (Fig. 2d).

These findings, taken together, suggest a correlation between mC and gene expression depending on the methylation context. Indeed, we identified a positive correlation between global transcript expression levels and CG methylation in both species (*B. terrestris* r_s_ = 0.23 and *T. angustula* r_s_ = 0.24) but not with CW (CA—cytosine/adenine or CT—cytosine/thymine) methylation (*B. terrestris* r_s_ = 0.08 and *T. angustula* r_s_ = -0.07). Curiously, when we only used the set of differentially expressed superTranscripts, no correlation was found between gene expression and mC in *B. terrestris*, neither at the CG (r_s_ = 0.08) nor at the CW (r_s_ = − 0.06) context. However, in *T. angustula*, both types of methylation presented negative correlations with gene expression in this scenario (CG r_s_ = − 0.31; CW r_s_ = − 0.35). This result suggests that DNA methylation indeed plays a role in subcaste task division of other eusocial bee species, as in honeybees, but in a more complex way than previously recognized.

### Comparative analyses of genes involved in task division among species

In order to recognize shared molecular mechanisms, different strategies were used. First, we asked whether the same genes were commonly involved in the observed subcaste differences of *A. mellifera*, *B. terrestris* and *T. angustula*. For *A. mellifera*, the list of genes differentially expressed between nurses and foragers was obtained from a previous study in which samples from the head, thorax and abdomen of these bees were analyzed separately^32^. When we compared our full-body transcriptomic data to each of these *A. mellifera* body parts, a significant number of genes were commonly differentially expressed (Table 1; Supplementary Tables SV–SVII). Among all three species, five genes were commonly differentially expressed when compared to the *A. mellifera* head, 5 when compared to the thorax and 4 when compared to the abdomen (Table 2).

**Table 1:** Number of genes in common among the set of differentially expressed genes between nurses and foragers of *B. terrestris*, *T. angustula* and *A. mellifera* samples. Overlap *p *value of significance from random sampling is shown; significant overlaps are indicated in bold.

		All DEG	*p *value	Nurses	*p *value	Foragers	*p *value
*A. mellifera* head and	*B. terrestris*	**42**	0.004	7	0.2208	15	0.1234
	*T. angustula*	**21**	0	7	0.0143	3	0.0474
*A. mellifera* thorax and	*B. terrestris*	82	0.5314	15	0.8628	**39**	0.0045
	*T. angustula*	24	0.0206	12	0.0586	**7**	0.0021
*A. mellifera* abdomen and	*B. terrestris*	74	0.0865	17	0.2129	19	0.6664
	*T. angustula*	**21**	0.0037	8	0.1015	4	0.0727
*B. terrestris* and *T. angustula*		**15**	3.00E-04	**7**	1.00E-04	2	0.306

**Table 2:** Genes differentially expressed between nurses and foragers common across all the three species.

*A. mellifera* sample	Gene	Commonly biased
**Head**	*Basement membrane-specific heparan sulfate proteoglycan core protein*	**Nurses:** *A. mellifera*, *T. angustula*
	*Cytochrome c*	**Nurses:** *A. mellifera, B. terrestris*, *T. angustula*
	*Histone h3*	**Nurses:** *B. terrestris, T. angustula*
	*Mucin-2-like*	**Nurses:** *A. mellifera*, *T. angustula*
	*Cytochrome p450*	**Foragers:** *A. mellifera, B. terrestris*, *T. angustula*
**Thorax**	*Basement membrane-specific heparan sulfate proteoglycan core protein*	**Nurses:** *A. mellifera*, *T. angustula*
	*Putative fatty acyl-coa reductase cg5065*	**Foragers:** *A. mellifera*, *T. angustula*
	*Cathepsin l*	**Nurses:** *A. mellifera, B. terrestris*, *T. angustula*
	*Cytochrome p450*	**Foragers:** *A. mellifera, B. terrestris*, *T. angustula*
	*Targeting protein for xklp2*	**Nurses:** *B. terrestris*, *T. angustula*
**Abdomen**	*Basement membrane-specific heparan sulfate proteoglycan core protein*	**Nurses:** *A. mellifera*, *T. angustula*
	*Histone h3*	**Nurses:** *B. terrestris, T. angustula*
	*Mucin-2-like*	**Nurses:** *A. mellifera*, *T. angustula*
	*Putative fatty acyl-coa reductase cg5065*	**Foragers:** *A. mellifera*, *T. angustula*

**Figure 4:**
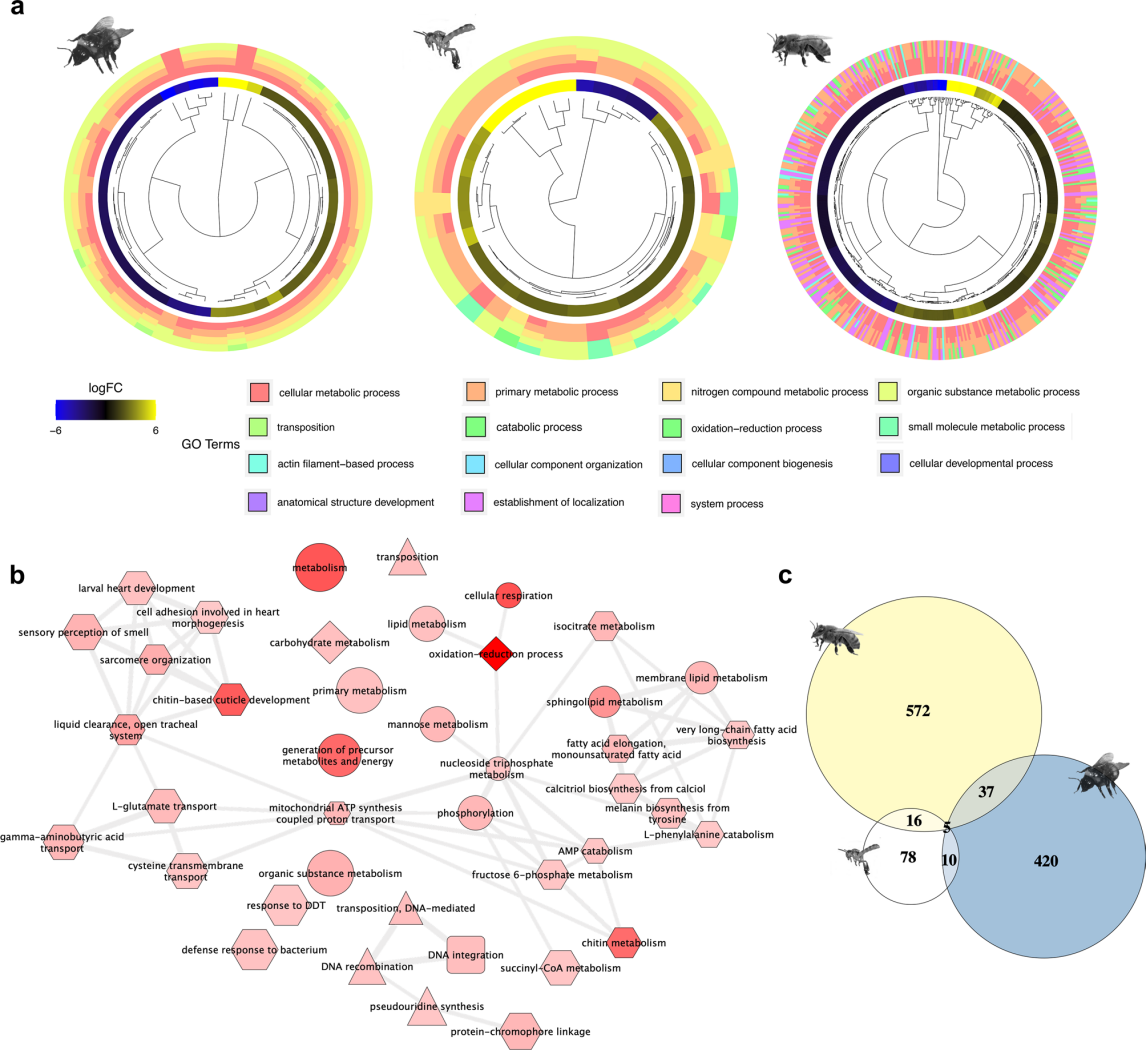
Comparisons among *B. terrestris*, *T. angustula* and *A. mellifera* head GO processes involved in task specialization. **a—**Hierarchical clustering of the differentially expressed transcripts using the third hierarchical level of GO annotation organized by their mean logFC difference between nurses and foragers. Outer circle colors indicate which GO term the gene could be associated. **b**—Similarity network of the enriched GO terms in all species, after semantic similarity-based reduction. GO terms that are more similar to each other are linked, and the line width indicates the degree of similarity. Edge shape indicates whether the shown term is enriched in *A. mellifera* (hexagon), in *B. terrestris* (triangle), in *T. angustula* (circle), commonly enriched in *B. terrestris* and *T. angustula* (square), or commonly enriched in *A. mellifera* and *T. angustula* (diamond). Edge color intensity indicates the *p *value in the enrichment test (the darker the color tone, the smaller the *p *value). Edge size indicates the frequency of the GO term in the entire UniProt database. **c**—Euler diagram showing the number of genes in common between the set of differentially expressed genes of each species. *A. mellifera* by A. Wide, *T. angustula* by L. Costa—images reproduced with permission from the original authors.

Considering the different body part samples of *A. mellifera*, the head was the only one with a significant overlap with the other two species (Table 1; Fig. 4c). We identified 42 genes, in the head sample, that were common between *A. mellifera* and *B. terrestris* (*p* = 0.004, mean number of genes expected by chance 32.96, SD = 5.5), and 21 genes between *A. mellifera* and *T. angustula* (*p* = 0.00, mean number of genes expected by chance 6.54, SD = 2.5). These results suggest that expression differences in the head have a strong influence on the subcaste worker type (nurse vs. forager). Interestingly, the expression pattern of overlapping genes was not always the same (Table 1). Compared to the honeybee, only genes differentially expressed in the thorax were significantly forager biased; 39 genes upregulated in *A. mellifera* foragers were also upregulated in *B. terrestris* foragers (*p* = 0.005, mean number of genes expected 30.95, SD = 5.20), while 7 were commonly upregulated in *T. angustula* foragers (*p* = 0.002, mean number of genes expected 2, SD = 1.35). Concerning the comparison between *B. terrestris and T. angustula*, 15 common genes were differentially expressed (*p* = 3e−04, mean number of genes expected by chance 7.06, SD = 2.6), and the upregulated genes in the nurses presented the most significant overlap (*p* = 1e−04, 7 overlapping genes, mean number of genes expected 2.16, SD = 1.45).

Secondly, we investigated whether the same molecular pathways could be involved in the task division of the three species. To address this possibility, we searched for similarities among the biological processes to which the differentially expressed genes were related and used a comparative approach based on GO subgraphs of the enriched terms. This type of analysis relies on the hierarchical graphical structure among the GO terms, where parent terms are more general and less specialized than child terms^42,43^. It has been reported that the use of subgraphs allows researchers to compare not only the enriched terms but also hierarchical connections, consequently reducing gene annotation bias^44^.

We performed a new GO enrichment test on differentially expressed transcripts of *A. mellifera*. For this comparison we used the functional annotation of biological processes from the *A. mellifera* genome, available at the Hymenoptera Genome Database^45^. The list of all enriched terms reported for *A. mellifera* is presented in Supplementary Table SII. Since the head showed the most significant overlap with our datasets, we used the enriched terms in this body part for the GO comparison (Supplementary Figures S3–S6).

We found that the enriched GO terms of all species were associated. For example, in *B. terrestris* and *T. angustula*, nearly all of the differentially expressed genes were nested under two main processes (Supplementary Figure S3): “metabolic process” (GO:0008152) and “cellular process” (GO:0009987). Notably, although specific enriched terms were distinct in both species (only “DNA integration” was commonly enriched), this divergence disappears at the parental levels of the topology, and almost all of the terms in the *B. terrestris* subgraph were also present in the *T. angustula* subgraph. The *A. mellifera* subgraph was more complex (Supplementary Figure S6), reflecting its more complete gene annotation. Still, among the top GO terms were the same GO processes identified in the nurse-forager differences (Supplementary Figure S3).

At the third hierarchical level, more lineage-specific GO processes start to emerge, such as “transposition” (GO:0032196) in *B. terrestris* and “catabolic process” (GO:0009056) in *T. angustula* (Fig. 4a). Nevertheless, genes showing the most significant differences in expression within species (i.e., higher absolute mean logFC between nurses and foragers) are usually those not related to these species-specific processes. This pattern is more evident in *B. terrestris* and *T. angustula* but is also observable in *A. mellifera* (Fig. 4a). The connection between the enriched GO terms in the set of differentially expressed genes of all species can also be visualized using semantic similarity-based clusters, as shown in Fig. 4b. This type of analysis reveals that the enriched GO terms of one species are frequently associated with the enriched GO terms of other species.

Since the methodology used to generate the *A. mellifera* datasets was slightly distinct^32^ from the approach used to generate *B. terrestris* and *T. angustula* data, and because numerous other studies have employed *Apis* to investigate the gene expression differences between nurses and foragers, we also reviewed the literature about the genes and molecular pathways commonly highlighted across studies. These comparisons are summarized in Box 1.

**Box 1:** Genes and molecular pathways commonly described in the literature as being involved in honeybee worker task division compared to present findings in *B. terrestris* and *T. angustula*. For—foragers; Nur—Nurses. Symbols indicate whether evidence suggests that the expression is higher (↑) or lower (↓) in one group compared to the other. Blue indicates higher expression levels in foragers than in nurses and orange indicates the opposite; (≈) in black, no changes identified or controversial evidence; and (↑↓) in red, indicates a mixed pattern, with some genes in the pathway being upregulated or downregulated in one of the two subcastes. *A. mellifera* by A. Wide, *T. angustula* by L. Costa—images reproduced with permission from the original authors.

Juvenile hormone (JH)	These hormones are important regulators in honeybee maturation affecting the task division system in workers^46^. In honeybees, foragers have higher levels of JH than nurses^4,5,46^, but in primitively eusocial bees, changes in JH appear not to affect worker behaviour^37^. This observation led to the hypothesis that JH might only be involved with age-related task division^47,48^. In the present dataset, we did not find any direct evidence of the involvement of JH in the age-related task division of *T. angustula* workers. This result is in agreement with previous studies about JH in stingless bees, which demonstrated that JH expression differences are important in differentiating queens and workers but not nurses and foragers. Notably, significantly reduced JH titer levels in foragers have been reported^49^. One transcript in our dataset, highly expressed in* B. terrestris* foragers, was indirectly related to JH pathways and predicted to be a “takeout-like” gene. This gene family has been associated with multiple processes in insects, including eusocial insects, in which it has been shown to be strongly sensitive to queen pheromone^50^
*Vitellogenin* (*vg*)	This yolk precursor protein is related to egg production in many insects^51^. In honeybees, it interacts with JH in a double repressor network, and its expression is reduced in foragers^4,5,51^. For bumblebees, this double repressor network apparently does not exist; instead, this protein gene has been associated with worker aggression^37^ and reproductive status when expressed in the fat body^52^. Our *B. terrestris* data identified two highly expressed genes in foragers with *vg *transcription factor domains. As a primitively eusocial species, bumblebee workers may dispute reproductive status with queens in later stages of the colony cycle^53^. In this sense, it would be interesting to determine if the augmented expression of these vg associated genes in foragers could be related to this behavior. Similar to honeybees, we found a higher expression of one *vg* receptor gene in *T. angustula* nurses, indicating the relevance of this protein in this subcaste. It has been proposed that since stingless bee workers usually produce trophic eggs^54^, *vg *might be involved in this process or even have alternative and/or unknown roles^55^
*Foraging* (*for*)	This gene has been reported as highly expressed in honeybee^56^ and bumblebee^38^ foragers. In honeybees, although the gene expression of this gene was not among the best predictors of the subcaste division of workers ^5,7^, its association with foraging is well established in the literature^56,57^. In bumblebees, the results about its effects are more controversial^58^, as its expression was higher in nurses than foragers in one study^36^. In our datasets, this gene was not differentially expressed
*Period *(*per*)/circadian rhythm	The gene* period* is related to circadian rhythm and has been reported as overexpressed in honeybee foragers^59,60^. This specific gene does not appear among the ones differentially expressed in our study. However, *B. terrestris* foragers have other highly expressed rhythm genes such as *protein quiver *or* sleepless* that are related to sleep, rhythmic process, and regulation of circadian sleep/wake cycles. Conversely, none of the differentially expressed superTranscripts of *T. angustula* were associated with rhythm genes. This result suggests that in *B. terrestris*, and* Apis*, rhythm genes are more relevant to nurse/forager behavioral differences than in *T. angustula*
Insulin/Insulin-like signaling (IIS)	In bees and other insects, genes involved in this pathway are important regulators of metabolism and feeding-related behavior^58,61,62^. In *Apis mellifera*, this energetic pathway is related to the subcaste division of workers and with lipid storage (lower levels of lipid storage increase IIS gene expression)^61^. We identified differentially expressed genes between nurses and foragers in both species studied herein, and some were related to insulin metabolism (genes containing insulin domains, transcription factor and regulators). These observations, taken together, indicate that the regulation of the insulin signaling pathway is essential to worker subcaste specialization in all these eusocial bees
Energetic metabolism	In general, since feeding circuits are basal pathways to different bee activities, genes related to energetic metabolism are expected to be involved in worker bee behavior^58,63^. Indeed, many genes related to energetic metabolism are differentially expressed in nurses and foragers of both species, with some of the common GO enriched terms related to this pathway. Specific examples of genes involved in energetic pathways (besides JH and IIS) studied in honeybees include *malvolio* and major royal jelly proteins^64,65^. The first was not differentially expressed in our data, and the second was related to differentially expressed superTranscripts in* B. terrestris*. In *B. terrestris* nurses, two highly expressed genes were predicted as protein yellow genes (which have a major royal jelly protein family domain), and in foragers, two other overexpressed genes had major royal jelly protein family domains
Transcription factors (TF)	Different TFs are believed to be involved in the dynamic changes related to behavior in eusocial bees^62^. Indeed, we identified differentially expressed TF superTranscripts in both species. However, it should be pointed out that the *ultraspiracle* (*usp*) TF, which is known to participate in the honeybee worker task division transition via its interaction with JH^66^, was not among them
DNA methylation/epigenetic modifications	DNA methylation is known to participate in the nursing to foraging transition in honeybees^17,18^. In the two species investigated in the present study, genes possibly related to epigenetic changes were also differentially expressed. Histone genes (H3 and H2B) and a methyltransferase in *T. angustula* were differentially expressed, and histone H3-K4 demethylation was differentially expressed, and lncRNAs were detected in *B. terrestris*. Except for one lncRNA overexpressed in *B. terrestris* foragers, all of these genes were highly expressed in nurses

## Discussion

The present study sought to identify common, as well as species-specific differential gene expression patterns related to the molecular basis underlying worker task division across all eusocial lineages of corbiculate bees. Towards this goal, we evaluated the contribution of conserved and taxonomically restricted molecular mechanisms to the evolution of this behavior. It was found that most of the species-specific mechanisms were related to gene expression patterns. Many of the differentially expressed genes were not common to all species, and among the ones that were, the pattern of expression was not necessarily the same. In other words, genes highly expressed in one species subcaste were often down-regulated in the same subcaste of the other species.

For instance, genes related to the circadian rhythm are highly expressed in foragers of *B. terrestris* and *Apis*^59,60^, but not in *T. angustula* foragers. Moreover, genes involved in yolk production, such as the *vg*-related genes, are highly expressed in nurses of both *T. angustula* and *Apis* ^4,5,51^, but not in *B. terrestris* nurses. These discrepancies are not entirely unexpected since each lineage has undergone unique selective pressures, despite presenting similar behaviors^67^. Even closely related species (within the same taxonomic genus) are known to exhibit different expression patterns for certain genes^12^. Thus, the expression profile of particular genes in one single species, should not be directly extrapolated to explain the responses of other species.

A notable example of how such assumptions can be misleading is the *vg/*JH network, which has been primarily studied in honeybees. In this situation, honeybee nurses have higher levels of *vg* and lower levels of JH when compared to foragers. However, when worker bees become foragers, the JH levels increase, and *vg* levels decrease in a double repressor network^4,66^. On the other hand, as demonstrated previously in bumblebees^37^ and corroborated by our data, this network is not regulated in the same manner in other species. In the bumblebee, genes related to JH and *vg* were both highly expressed in foragers, and in *T. angustula,* we found evidence of *vg* being related to nursing behavior but did not observe the high expression of the JH genes in foragers. These results support the hypothesis that the typical *vg/*JH double repressor network observed in honeybees is not functional in stingless bees, and the *vg* is distinctly regulated^49,55^.

Despite these apparent differences, the gene expression dynamics in worker behavior are not completely unrelated among eusocial bees. Beyond the exact expression trend, we still found a significant number of common genes that were differentially expressed in nurses and foragers from all three species. Interestingly, common genes like *cytochrome p450*, *fatty acyl-CoA*, as well as some mitochondrial- and histone-related genes have also been shown to be responsive to queen pheromone in ants and bees^50^. Moreover, the enriched biological process terms associated with the differentially expressed superTranscripts from all three species were found to be very similar. Our comparisons of the enriched GO term subgraphs revealed broader similarities among *A. mellifera*, *B. terrestris* and *T. angustula* and illustrated how distinct GO terms (and genes) were involved in similar biological processes. In general, biological terms related to energetic and metabolic processes, including “organic substance metabolic process”, “primary metabolic process”, “nitrogen compound metabolic process” and “cellular metabolic process”, were central to subcaste differentiation in all species.

Over the years, the relevance of metabolic pathways to insect sociality has been demonstrated in many studies^30,63,68,69^, and it has become clear that this is not a species-specific trait. Indeed, these pathways are affected by queen pheromone in different species and are involved with caste determination of multiple hymenopteran lineages, including bees, ants and wasps^25,50^. Given the central role of energetic and metabolic maintenance in any living animal, it is not surprising that changes in these pathways will affect a variety of features, including behavioral phenotypes. However, in terms of gene regulation, it is fascinating to observe how plastic and dynamic these networks can be, with different lineages evolving individual responses to similar cues (like queen pheromone).

Regarding the evolutionary history of the differentially expressed genes, we detected an increased proportion of taxonomically restricted genes among the subcaste biased genes in *B. terrestris* and *T. angustula*. This observation highlights the relevance of new genes in the evolution of behavioral traits, as suggested previously^22,24,28^. However, the higher proportion of conserved genes among the ones differentially expressed, including genes from orthogroups common to all Apinae, cannot be overlooked. Similarly, Warner et al.^32^ showed that new genes, in pharaoh ants and honeybees, tend to represent a higher proportion of caste and behavioral biased genes, although ancient conserved pathways are also essential for caste differences. Additionally, these authors found that the transcription architecture associated with caste was much more conserved than subcaste specialization when comparing ants and bees^32^.

This mosaic pattern of species-specific features involved in common molecular processes is also observed in the epigenetic machinery. Transcriptomic and WBS data support the involvement of DNA methylation and other epigenetic factors in worker specialization of the two analyzed species. Among the differentially expressed genes, we detected genes involved in epigenetic alterations in all bees and observed that the global methylation patterns of *B. terrestris* and *T. angustula* were distinct from their differentially expressed superTranscripts. As shown in Figs. 2 and 3, the differentially expressed superTranscripts had less CG and more overall mC methylation. Nevertheless, a closer investigation revealed distinct epigenetic mechanisms in the two bees.

For instance, the epigenetic-related genes that are differentially expressed in each species are different. We also found that genes highly expressed in *T. angustula* foragers were more methylated at the CG context and had higher mean mC levels when compared to genes overexpressed in nurses. Interestingly, these methylation trends were found to be the opposite in *B. terrestris*. Based on the fact that the WBS data was obtained from nurses of both species, these results were entirely unexpected.

While DNA methylation was frequently observed at the CG context in *B. terrestris* and *T. angustula*, methylation at other nucleotide contexts (i.e., non-CG or non-CpG methylation) also occurred. Originally, non-CG DNA methylation was frequently associated with several processes in plants^70,71^, but its function in other eukaryotes has been gaining more attention^72^. Still, the effects of differential DNA methylation contexts in most organisms are poorly understood and underestimated (reviewed in^72,73^). Previous studies have demonstrated that methylation at CG and non-CG contexts are typically mediated by distinct mechanisms^74^, where CG methylation constitutively occurs via DNA methyltransferase 1 (Dnmt1)^72,73^ and non-CG methylation is maintained by de novo methylation mechanisms involving DNA methyltransferase 3 (Dnmt3)^75^. In this sense, non-CG methylation is mostly related to novel and more variable epigenetic alterations^73^. Supporting evidence for the existence of non-CG methylation in social insects was previously reported for ants^76^ and honeybees, especially in the head^75^. While non-CG methylation seemed to be involved with alternative mRNA splicing and it was especially enriched in genes previously related to behavioral responses in honeybees, no direct connection with sociality could be established^75^. Herein, we demonstrated evidence for such connection when it was shown that different proportions of CG and non-CG methylation were present in the set of differentially expressed superTranscripts when compared to the general transcriptomic profile.

Since the identification of functional Dnmt genes in the genomes of ants, bees and wasps, DNA methylation is now considered to be an important player in the epigenetic control of sociality (reviewed in ^15,77,78^). Given the relevance of DNA methylation in brain development and maturation in mammals, it seems likely that DNA methylation, along with other epigenetic mechanisms, could regulate the behavior of social insects^77,78^. This indeed was demonstrated in several studies using bees and ants^52,75,76,79^, and in honeybees, the knockout of Dnmt3 significantly affected gene splicing by exon skipping and intron retention^80^. Nonetheless, some conflicting results about the role of DNA methylation in caste differences have been reported in the literature^78^. For example, in *Polistes* wasps, it was shown that DNA methylation is not essential for the establishment of reproductive castes^26,81^. Intriguingly, the Dnmt3 coding gene was also not found in the *Polistes* genome^26,81^. As this enzyme participates in the establishment of de novo and non-CG methylation^75^, it seems reasonable to assume that Dnmt3 could at least partially mediate the link between DNA methylation and behavioral dynamics^26^. Our results indicate that both CG and non-CG methylation play a role in worker task division, supporting the hypothesis that Dnmt3 activity would be necessary in the worker specialization transition, as seen in corbiculates. Further data are necessary to infer how specific methylation contexts could affect certain behavioral changes and if these alterations are somewhat conserved across species. However, based on the results gathered so far, we hypothesize that non-CG methylation dynamics are relevant to task division in workers and possibly other social traits.

Higher levels of mC in bees have been associated with an increase in gene expression, i.e., genes with more methylation also have higher expression levels^15^. In the present study, this correlation was observed for CG methylation in both species tested, but not for methylation at the non-CG context. In fact, among the differentially expressed superTranscripts of *T. angustula*, where higher levels of non-CG methylation are observed, we found a negative correlation between gene expression and DNA methylation. This observation suggests that the effect of mC in bee gene expression might differ according to the methylation context; CG methylation seems to increase gene expression while non-CG methylation might suppress it. However, the exact effect of DNA methylation nucleotide context and genomic location in gene expression is an open debate^73,76,82,83^. In mammals, CG methylation in promoter regions suppresses gene expression while gene body CG methylation is more complex^84^, but it is generally associated with increased gene expression^73^.

On the other hand, non-CG methylation is highly tissue and cell type-specific, and its correlation with gene expression is unclear^83,85^ as it seems to depend upon the genomic context in which it occurs (reviewed in ^83^). One of the possible mechanisms through which non-CG methylation affects gene expression is recruiting the methyl-CpG-binding protein (MeCP2)^86^. This protein is a transcriptional repressor, and its interaction with non-CG methylated sites might explain the negative correlation between gene expression and non-CG methylation observed in neurons^83^. This mechanism of gene expression suppression demonstrates how non-CG methylation may negatively affect gene expression levels, as observed in our analyses.

Finally, it is important to consider some of the limitations of the present study. First, aiming to obtain a global overview of gene expression and DNA methylation differences, we used full bodies for the transcriptomic and bisulfite sequencings. Since we know that different body parts, tissues and even cells have unique gene expression dynamics^13^, our approach likely reduced our ability to detect small scale alterations and specific methylation contexts. Nonetheless, our comparative analyses with specific body parts from *A. mellifera* demonstrated that the full-body RNASeq data still detected gene expression differences significantly comparable to the head and other tissues, thus providing an overall perspective of the differences between nurses and foragers, as expected. Moreover, to facilitate the comparisons between *B. terrestris* and *T. angustula*, we employed similar pipelines in the analyses of both species. Consequently, we occasionally compromised the bumblebee analysis to match it with the dataset from the species with no reference genome available. For example, we annotated both species transcriptomes based on search similarities to databases instead of using the *B. terrestris* genome for its annotation. In this sense, our approach may have affected the GO enrichment analysis. Additionaly, differently from genome annotation, transcriptomic annotation is redundant, i.e., multiple transcripts (or superTranscripts in our case) may annotate to the same gene, and this affects the frequency of the GO terms in the dataset. To deal with this, we kept the frequency of GO terms proportional in the enrichment test by using the appropriate background list (in our case, the complete transcriptome set), which is the recommended approach for GO enrichment tests^87^. Despite these efforts, there is still a possibility that the chosen approach biased our enrichment statistics.

Nevertheless, since GO annotations are dynamic and always biased by database representation^88^, we chose to apply the same methodological approach to both species. In this manner, if the enrichment test is biased, it will be equally biased in both species. Finally, we did not validate our gene expression results with an alternative, independent method (such as real-time reverse polymerase chain reaction). Given due consideration, the present study can only describe broad patterns and conclusions regarding the general species expression and methylation profiles. Thus, future studies attempting to detect more subtle and detailed differences are necessary.

In the present study, we provided valuable insights into social behavior evolution. Our datasets aligned with the honeybee literature, allowed us to compare all of the eusocial corbiculate bee groups: Apini, Bombini and Meliponini. The main findings support a complementary role for conserved and new genes in subcaste differences. In our analyses, the toolkit hypothesis is sustained by the existence of common and more ancient molecular mechanisms involved in worker task division across these species, standing as central among them energetic and metabolic pathways, and epigenetic factors. However, despite these similarities, particular gene expression patterns tend to be species-specific, and an increased proportion of subcaste biased genes were found to be taxonomically restricted, corroborating the new gene hypothesis.We conclude that this scenario could be explained by more recent specialization of species-specific molecular responses to ancient social cues, consequently leaving a mosaic profile of the worker task division, where both unique and shared features are observed.

Given that worker specialization is a very plastic and environmentally responsive behavior in eusocial bees^10,18^, we expect that this behavior is regulated by an even more substantial proportion of species-specific elements when compared to less responsive traits in social insects, such as caste differences^32^. Moreover, our results indicate that non-CG methylation is relevant to worker behavioral dynamics in eusocial corbiculates and that it might affect gene expression differently from CG methylation. As a result, the involvement of non-CG methylation in eusociality should be further investigated.

## Material and methods

### Sample collection and sequencing

Bee species were chosen based on their behavior (primitively eusocial and highly eusocial), phylogenetic relationship (corbiculate bees^39^), and sampling convenience. Samples were collected from three separate colonies of each species. The *B. terrestris* colonies were obtained from a commercial supplier (Biobest) and were maintained under lab conditions at Queen Mary University of London (England). All of the bees in the colonies were marked and housed in wooden boxes attached to foraging arenas, only individuals emerged after colony transfer were sampled. After 16 days of adaptation, all recently born workers received an individual number tag. Since bumblebee workers do not usually forage following a stressful situation or emergency^89^, we waited five additional days before starting the sampling. Concerning *T. angustula*, colonies regularly maintained in wooden boxes at the Laboratório de Abelhas (University of São Paulo—Brazil) were used for sample collection. These colonies were orginary from different locations of the São Paulo state, and they were allowed to forage and breed freely within the university campus—where this species is native and abundant—for at least three months before sampling, therefore their relatedness level is uncertain.

Worker subcastes were determined using two different approaches. For *B. terrestris*, colonies were observed for one day during all their active foraging period (6 h uninterrupted) and tagged bees that never entered the foraging arena and remained inside the nest during the entire period were considered nurses. On the following day, foragers were collected first, while collecting nectar in the foraging arena, and then nurses were collected inside of the colonies. All of the collected samples were immediately frozen in liquid nitrogen. For *T. angustula*, nurses were defined by age. Briefly, brood cells (from which adults were about to emerge) were removed from the colonies and transferred to a temperature- and humidity-controlled incubator. Upon emergency, female workers were marked with specific colors using water-based ink and immediately returned to the colony. Ten to twelve days after their emergency and reintroduction, colonies were opened, and marked individuals were collected. During this age, worker bees from *T. angustula* present nursing behavior^54^. Foragers were collected while leaving and returning to the colonies from foraging trips. To prevent collecting guard workers^2^, we avoided the bees standing in front of the colony entrance. It should be pointed out that some foragers were collected before and after the nurses, but none were collected while the nurses were being marked and collected. This approach was employed to avoid colony disturbance effects in the behavior of the workers. Nurses from different colonies were collected on different days.

For both species, all individuals were sampled between 10–12 h, and the entire bodies of the workers were used for RNA and DNA extraction. For RNA-Seq, six *T. angustula* workers, from the same colony and subcaste, were pooled as one sample, and three *B. terrestris* workers per subcaste/colony were pooled as one sample. Each colony was considered as one sample replicate. Total RNA was extracted from workers using the Qiagen extraction kit (RNeasy Mini Kits). RNA quality and quantification were verified spectrophotometrically using a Bionalyzer, Nanodrop and/or Qubit. RNA sequencing was performed on an Illumina HiSeq 2000, and the sequencing providers performed the library preparation. *B. terrestris* workers were sequenced by the Genome Center at Queen Mary University of London, and *T. angustula* samples were sequenced at LACTAD (Unicamp). RNA sequencing generated 30–50 million paired reads (100 bp) per colony replicate. For whole bisulfite sequencing (WBS), one nurse (whole-body) per species was used for the phenol–chloroform DNA extraction^90^. The WBS was performed following the protocol described in^91^ using an Illumina NextSeq500. Sequencing and library preparation were performed at the University of Georgia. In total, the WBS returned 60–70 million single reads (150 bp) per sample, and all sequenced reads are available at BioProject ID PRJNA615177. All of the sampling and experimental procedures were in accordance with the relevant local guidelines and regulations, and no committee approval was necessary.

### Transcriptome assembly and differential expression analyses and comparisons

Read quality assessments were performed using the FastQC program (v0.11.2)^92^ before and after cleaning. The FASTX Toolkit (v0.0.14)^93^ was used to trim the first 14 bp of all reads because an initial GC bias^94^ was detected. Low-quality bases (phred score below 30) and small reads (less than 31 bp) were removed using SeqyClean (v1.9.3)^95^. Samples from nurses and foragers were combined for the assemblies. We then digitally normalized (20 × coverage) the cleaned reads to increase de novo transcriptome assembly efficiency^96^. Transcriptome assembly was performed differently for each species. For *B. terrestris*, its genome^97^ was used as a reference by two approaches. First, using HISAT2 (v2-2.0.3)^98^ and StringTie (v1.2.2)^99^, a regular reference assembly was obtained. Secondly, the Trinity (v2.1.1)^100^ program was used to perform a reference guided de novo assembly. The two resulting assemblies were merged using CD-Hit (v4.6)^101^, Corset (v1.05)^102^ and Lace (v0.80)^40^ to cluster transcripts into superTranscripts. We have chosen to use this combined approach for *B. terrestris* for two reasons. First, to optimize the transcriptome assembly based on our dataset, a recommended procedure even for species with well-annotated reference genome and transcriptome^103^. Second, to make *B. terrestris* and *T. angustula* datasets more comparable since, for the latter, we have used the clustering method. There is no reference genome for *T. angustula*; therefore, we performed a combined de novo assembly using two strategies with the Trinity pipeline: a reference guided de novo assembly, based on the genome of another stingless bee, *Melipona quadrifasciata*^104^; and a complete de novo assembly. Afterward, the two assemblies were merged as in the bumblebee. Assemblies used the default recommended parameters of the programs. CD-Hit was used to merge transcripts with more than 95% similarity, Corset was set to keep transcripts with a minimum of 50 × coverage, and Lace was used to obtain the superTranscripts.

SuperTranscripts were then annotated with Annocript (v1.2)^105^ using the UniProt Reference Clusters (UniRef90) and the UniProtKB/Swiss-Prot databases^106^ (June 2016 version). SuperTranscripts with significant blast hits (e-value < 1e−5) against possible contaminants (plants, fungus, mites and bacteria) in the UniRef90 were removed from the final datasets. Finally, only potentially coding superTranscripts (based on blast results and ORF analysis) or possible lncRNAs were kept. This annotation pipeline was used for both species. Quality parameters from the transcriptomes were analyzed using QUAST (v4.0)^107^, BUSCO (v2)^108^ and Qualimap (v2.2)^109^.

Differential expression analyses were performed in each species independently and compared afterward, as illustrated in Supplementary Figure S7. Bowtie2 (v2.2.5)^110^, RSEM (v1.2.22) ^111^ and DESeq2^112^ (*p* value < 1e−3) were used to identify differentially expressed superTranscripts, using scripts from the Trinity package—only the figure parameters were adapted. During the analyses, we identified a possible batch effect in samples from *T. angustula*: one nurse and one forager replicate were sequenced in different lanes, and it seemed to affect sample correlation. This effect was corrected during the differential expression analyses following the suggested protocol in the DESeq2 documentation. No batch effect was identified in *B. terrestris* samples. The *A. mellifera* differential expression results were obtained from^32^. To test whether any GO term was enriched in a set of differentially expressed superTranscripts compared to the total transcriptome, a classical Fisher’s exact test was performed using the R package TopGO^44^. The GO enrichment analyses for the honeybee differentially expressed genes were performed as for the other species except that we used a weighted Fisher’s exact test. GO terms were obtained from the Amel_HAv3.1 functional annotation of biological processes available at the Hymenoptera Genome Database^45^ (accessed in July 2020). We used the NCBI gene information from *A. mellifera* (NCBI: txid7460) to overlap gene id and GO annotation. The background gene set of the GO terms was the genes used for the differentially expressed analysis in^32^. For the comparative figures of subgraphs, we used the subgraph induced by the top 8 enriched terms for *A. mellifera*.

Species comparisons of differentially expressed genes were based on gene annotation, only using unique and non-redundant terms (i.e., those genes not containing “uncharacterized protein” in their annotation). The list of overlapping genes was then manually curated to remove annotation incoherencies not detected computationally, e.g., when gene lists from *B. terrestris* and *T. angustula* were compared with our R script, 18 terms were common. After manual curation, we removed three genes from this list because of partial or redundant annotation matches ("transposase", "transporter" and "cytochrome c oxidase subunit [fragment]”), leaving 15 genes in common. In the random sampling statistics, this manual filtering correction was not used, so the numbers of common genes obtained with the computational comparison were used. Comparisons between the set of GO enriched terms and subgraphs were performed manually. The similarity network parameters were estimated with REVIGO^113^ and applying the medium (0.7) similarity threshold. In the interactive network mode of this program, the input data for Cytoscape^114^ was downloaded for further figure editing. Statistical tests of significance for comparisons were based on random sampling using R scripts^115^, and *p *values of less than 0.01 were considered significant. The utilized scripts are available at https://github.com/nat2bee/Foragers_vs_Nurses.

### Taxonomically restricted genes analysis

Transcriptome ORFs were predicted for the superTranscripts of *B. terrestris* and *T. angustula* using TransDecoder (v5.5.0)^116^. Predicted amino acid sequences were then compared to the proteins annotated from the genomes of nine other Apinae species (*Apis cerana*—assembly ACSNU-2.0^117^, *Apis mellifera*—assembly Amel_HAv3.1^118^, *Bombus impatiens*—assembly BIMP_2.2^119^, *Bombus terrestris*—assembly Bter_1.0, *Euglossa dilemma*—assembly Edil_v1.0^120^*, Eufriesea mexicana*—assembly ASM148370v1^104^, *Frieseomelitta varia*—assembly Fvar_1.2^121^, *Melipona quadrifasciata*—assembly ASM127656v1 and *Habropoda laboriosa*—assembly ASM126327v1^104^) using OrthoFinder (v2.3.12)^41^ to obtain the orthogroups of the bees. The identification of orthogroups within our defined categories [apinae, corbiculates, social corbiculates, bumblebees, bterrestris (G), stingless bees, stingless bees (F) and species-specific] was based on filtering the genes/orthogroups table (Supplementary Information S6).

### DNA methylation analysis

Cleaning and adapter trimming of the bisulfite-converted reads were performed with Trim Galore (v 0.4.3)^122^ wrapper script using the default parameters. Since the coding regions are the main methylation targets in bees and other Hymenoptera^15^, we used the complete transcriptome assemblies as the reference when analyzing DNA methylation. PCR bias filtering, cleaned read alignment and methylation call were performed using the BS-Seeker2 (v 0.4.3)^123^. Notably, this program employs Bowtie2 in the local alignment mode, which is necessary for properly aligning the WBS reads to a transcriptome. CGmapTools (v 0.0.1)^124^ was used to filter low coverage methylated sites (< 10 ×) and to obtain DNA methylation statistics, including context use. Remaining statistical tests were performed using R, as follows: a random sampling test was used to verify whether the proportion of CG methylation found deviated from what was expected by chance; a one-tailed z-test was used to determine whether differences between the mean methylation observed in the set of superTranscripts was different from the general transcriptomic mean; the correlation between methylation and gene expression was calculated using Spearman’s correlation coefficient between the superTranscript mean methylation and its normalized read count. The utilized scripts are available at https://github.com/nat2bee/Foragers_vs_Nurses.

## Supplementary information

Supplementary Information 1.

Supplementary Information 2.

Supplementary Information 3.

Supplementary Information 4.

Supplementary Information 5.

Supplementary Information 6.

## Data Availability

The datasets generated during the current study are available either in the NCBI repository [BioProject ID PRJNA615177] or in the project repository at GitHub [https://github.com/nat2bee/Foragers_vs_Nurses].
