# Supplementary material for: Gene expression and epigenetics reveal species-specific mechanisms acting upon common molecular pathways in the evolution of task division in bees
Source: Sci Rep. 2021 Feb 11;11:3654. doi: 10.1038/s41598-020-75432-8 (PMC7878513; doi:10.1038/s41598-020-75432-8)
Supplement: Supplementary file 1 — Supplementary Information 1. [file 41598_2020_75432_MOESM1_ESM.pdf]

Gene expression and epigenetics reveal species-specific mechanisms acting upon common molecular pathways in the evolution of task division in bees

Natalia de Souza Araujo[1,2] and Maria Cristina Arias[1]

[1] Department of Genetics and Evolutionary Biology, Universidade de São Paulo. Rua do Matão, 277, 05508-090 São Paulo - SP, Brazil

[2] [current address] Interuniversity Institute of Bioinformatics in Brussels and Department of Evolutionary Biology & Ecology, Université Libre de Bruxelles. Avenue F.D. Roosevelt, 50. B-1050 Brussels, Belgium

S1 - Supplementary File 1

Supplementary Tables

Table SI Main quality parameters from the complete transcriptome assembly of superTranscripts from *T. angustula* and *B. terrestris* workers. Annotated superTranscripts refer to superTranscripts with at least one significant blast hit against the UniRef90 database (e-value <1e-5).

	T. angustula	*B. terrestris*
Total Number of superTranscripts	33,065	27,987
N50	3,614	5,490
%GC	38.11	37.19
Mean coverage per sample	≅100x	≅130x
Annotated superTranscripts	26,623	21,638
Number of lncRNA	347	431
BUSCO stats (n: 4,415)	C:86.2% [S:49.2%, D:37.0%] F:7.9%, M:5.9%	C:91.9% [S:47.5%, D:44.4%] F:3.9%, M:4.2%

Table SII Complete list of Gene Ontology terms of Biological processes enriched among the differentially expressed genes in *A. mellifera* body parts, *B. terrestris* and *T. angustula*. Significance was obtained using Fisher's exact enrichment test (p <0.01).

			B. terrestris		
**GO ID**	**GO Term**	**Annotated**	**Significant**	**Expected**	**p-value**
GO:0006313	transposition, DNA-mediated	29	5	1.09	0.0039
GO:0032196	transposition	29	5	1.09	0.0039
GO:0015074	DNA integration	139	12	5.23	0.0049
GO:0006310	DNA recombination	31	5	1.17	0.0053
GO:0001522	pseudouridine synthesis	4	2	0.15	0.008
			T. angustula		
GO:0045333	cellular respiration	72	7	0.49	4.30E-07
GO:0008152	metabolic process	3868	41	26.24	5.70E-07
GO:0006091	generation of precursor metabolites and energy	136	8	0.92	2.90E-06
GO:0022900	electron transport chain	35	5	0.24	3.20E-06
GO:0055114	oxidation-reduction process	138	8	0.94	3.20E-06
GO:0015980	energy derivation by oxidation of organic compounds	102	7	0.69	4.60E-06
GO:0005975	carbohydrate metabolic process	379	12	2.57	5.10E-06
GO:0009060	aerobic respiration	51	5	0.35	2.10E-05
GO:0022904	respiratory electron transport chain	27	4	0.18	2.90E-05
GO:0006119	oxidative phosphorylation	30	4	0.2	4.40E-05

GO:0006665	sphingolipid metabolic process	14	3	0.09	0.0001
GO:0019646	aerobic electron transport chain	6	2	0.04	0.00066
GO:0071704	organic substance metabolic process	3675	35	24.93	0.00125
GO:0006643	membrane lipid metabolic process	38	3	0.26	0.00208
GO:0016310	phosphorylation	137	5	0.93	0.00219
GO:0006013	mannose metabolic process	11	2	0.07	0.00238
GO:0006122	mitochondrial electron transport, ubiquinol to cytochrome c	12	2	0.08	0.00284
GO:0015074	DNA integration	48	3	0.33	0.00407
GO:0046034	ATP metabolic process	98	4	0.66	0.00419
GO:0006629	lipid metabolic process	398	8	2.7	0.00472
GO:0042775	mitochondrial ATP synthesis coupled electron transport	16	2	0.11	0.00508
GO:0044238	primary metabolic process	3416	32	23.18	0.00517
GO:0009144	purine nucleoside triphosphate metabolic process	107	4	0.73	0.00573
GO:0009205	purine ribonucleoside triphosphate metabolic process	107	4	0.73	0.00573
GO:0000270	peptidoglycan metabolic process	17	2	0.12	0.00574
GO:0006027	glycosaminoglycan catabolic process	17	2	0.12	0.00574
GO:0009253	peptidoglycan catabolic process	17	2	0.12	0.00574
GO:0009199	ribonucleoside triphosphate metabolic process	112	4	0.76	0.00673
GO:0009141	nucleoside triphosphate metabolic process	114	4	0.77	0.00716
GO:0042773	ATP synthesis coupled electron transport	20	2	0.14	0.00791
***A. mellifera* - head**					
GO:0055114	oxidation-reduction process	472	85	39.08	4.20E-10
GO:0006099	tricarboxylic acid cycle	24	12	1.99	9.90E-08
GO:0040003	chitin-based cuticle development	104	27	8.61	9.90E-07
GO:0006030	chitin metabolic process	50	17	4.14	3.90E-06
GO:0035002	liquid clearance, open tracheal system	18	7	1.49	3.60E-04

GO:0045214	sarcomere organization	43	11	3.56	9.70E-04
GO:0035159	regulation of tube length, open tracheal...	36	10	2.98	1.43E-03
GO:0007608	sensory perception of smell	129	14	10.68	1.55E-03
GO:0042776	mitochondrial ATP synthesis coupled proton transport	4	3	0.33	2.12E-03
GO:0018298	protein-chromophore linkage	4	3	0.33	2.12E-03
GO:0015812	gamma-aminobutyric acid transport	4	3	0.33	2.12E-03
GO:0006102	isocitrate metabolic process	4	3	0.33	2.12E-03
GO:0006723	cuticle hydrocarbon biosynthetic process	4	3	0.33	2.12E-03
GO:0034625	fatty acid elongation, monounsaturated fatty acid	13	5	1.08	2.80E-03
GO:0034626	fatty acid elongation, polyunsaturated fatty acid	13	5	1.08	2.80E-03
GO:0015813	L-glutamate transmembrane transport	13	5	1.08	2.80E-03
GO:0019367	fatty acid elongation, saturated fatty acid	13	5	1.08	2.80E-03
GO:0022900	electron transport chain	106	16	8.78	4.48E-03
GO:0005975	carbohydrate metabolic process	212	28	17.55	4.69E-03
GO:0006583	melanin biosynthetic process from tyrosine	5	3	0.41	4.97E-03
GO:0007508	larval heart development	5	3	0.41	4.97E-03
GO:0035151	regulation of tube size, open tracheal system	51	15	4.22	5.40E-03
GO:0042742	defense response to bacterium	151	16	12.5	5.62E-03
GO:1903712	cysteine transmembrane transport	2	2	0.17	6.84E-03
GO:0006002	fructose 6-phosphate metabolic process	2	2	0.17	6.84E-03
GO:0070777	D-aspartate transport	2	2	0.17	6.84E-03
GO:0060415	muscle tissue morphogenesis	2	2	0.17	6.84E-03
GO:0006196	AMP catabolic process	2	2	0.17	6.84E-03
GO:0006104	succinyl-CoA metabolic process	2	2	0.17	6.84E-03
GO:0046680	response to DDT	2	2	0.17	6.84E-03
GO:0042761	very long-chain fatty acid biosynthetic process	16	5	1.32	7.73E-03

GO:0006559	L-phenylalanine catabolic process	6	3	0.5	9.33E-03
GO:0061343	cell adhesion involved in heart morphoge...	11	4	0.91	9.57E-03
GO:0036378	calcitriol biosynthetic process from cal...	11	4	0.91	9.57E-03
***A. mellifera* - thorax**					
GO:0032543	mitochondrial translation	95	70	23.95	5.90E-20
GO:0006120	mitochondrial electron transport, NADH to ubiquinone	30	22	7.56	4.10E-08
GO:0061077	chaperone-mediated protein folding	30	19	7.56	2.80E-07
GO:0055114	oxidation-reduction process	472	200	119.01	8.20E-07
GO:0015986	ATP synthesis coupled proton transport	14	14	3.53	1.00E-06
GO:0006099	tricarboxylic acid cycle	24	16	6.05	2.20E-05
GO:0010998	regulation of translational initiation by eIF2 alpha phosphorylation	10	8	2.52	4.40E-04
GO:0008340	determination of adult lifespan	140	53	35.3	5.70E-04
GO:0002181	cytoplasmic translation	123	45	31.01	8.30E-04
GO:0006122	mitochondrial electron transport, ubiquinol to cytochrome c	14	9	3.53	2.27E-03
GO:0006123	mitochondrial electron transport, cytochrome c to oxygen	10	7	2.52	3.67E-03
GO:0042776	mitochondrial ATP synthesis coupled proton transport	4	4	1.01	4.03E-03
GO:0006108	malate metabolic process	4	4	1.01	4.03E-03
GO:0006094	gluconeogenesis	8	6	2.02	4.81E-03
GO:0042744	hydrogen peroxide catabolic process	6	5	1.51	4.81E-03
GO:0033617	mitochondrial cytochrome c oxidase assembly	13	8	3.28	5.93E-03
GO:0050829	defense response to Gram-negative bacterium	76	30	19.16	5.95E-03
GO:1904262	negative regulation of TORC1 signaling	11	7	2.77	7.91E-03
GO:0045214	sarcomere organization	43	19	10.84	9.19E-03
***A. mellifera* - abdomen**					

GO:0055114	oxidation-reduction process	472	123	91.29	1.10E-04
GO:0045214	sarcomere organization	43	20	8.32	4.40E-04
GO:0006636	unsaturated fatty acid biosynthetic process	10	7	1.93	0.00101
GO:0014009	glial cell proliferation	15	5	2.9	0.0014
GO:0002347	response to tumor cell	7	5	1.35	0.00398
GO:0007523	larval visceral muscle development	5	4	0.97	0.0059
GO:0009253	peptidoglycan catabolic process	5	4	0.97	0.0059
GO:0006030	chitin metabolic process	50	20	9.67	0.00656
GO:0036065	fucosylation	10	5	1.93	0.00721
GO:0010797	regulation of multivesicular body size involved in endosome transport	3	3	0.58	0.00722
GO:0035999	tetrahydrofolate interconversion	3	3	0.58	0.00722
GO:0034553	mitochondrial respiratory chain complex II assembly	3	3	0.58	0.00722
GO:0043508	negative regulation of JUN kinase activity	3	3	0.58	0.00722
GO:0040003	chitin-based cuticle development	104	34	20.11	0.00829
GO:0097502	mannosylation	18	6	3.48	0.00896
GO:0050830	defense response to Gram-positive bacterium	14	8	2.71	0.00974
GO:0061327	anterior Malpighian tubule development	11	6	2.13	0.0098

Table SIII Orthogroups identified using OrthoFinder, in bold are highlighted our transcriptomic data.

	A. cerana	*A. mellifera*	*B. impatiens*	***B. terrestris transcriptome***	*B. terrestris*	*E. dilemma*	*E. mexicana*	*F. varia*	*M. quadrifasciata*	***T. angustula transcriptome***	*H. laboriosa*
Number of genes	20,933	23,471	24,471	**32,428**	22,091	15,904	15,659	11,127	14,257	**37,396**	12,256
Number of genes in orthogroups	20,669	23,321	24,239	**29,116**	21,939	11,788	15,482	10,596	10,956	**29,408**	12,140
Number of unassigned genes	264	150	232	**3,312**	152	4,116	177	531	3,301	**7,988**	116
Percentage of genes in orthogroups	98.7	99.4	99.1	**89.8**	99.3	74.1	98.9	95.2	76.8	**78.6**	99.1
Percentage of unassigned genes	1.3	0.6	0.9	**10.2**	0.7	25.9	1.1	4.8	23.2	**21.4**	0.9
Number of orthogroups containing species	9,926	9,651	10,015	**12,140**	10,022	9,880	9,598	9,340	9,272	**12,660**	9,436
Percentage of orthogroups containing species	59.8	58.1	60.3	**73.1**	60.4	59.5	57.8	56.3	55.8	**76.3**	56.8
Number of species-specific orthogroups	64	35	42	**1,153**	23	109	38	37	64	**1,944**	19
Number of genes in species-specific orthogroups	173	132	118	**3,138**	58	1,116	97	90	351	**5,662**	67
Percentage of genes in species-specific orthogroups	0.8	0.6	0.5	**9.7**	0.3	7.0	0.6	0.8	2.5	**15.1**	0.5

Table SIV Number and proportion of orthogroups occurring in the different taxonomic classes.

**Orthogroup classification**	**all orthogroups**	**all orthogroups %**	***B. terrestris* transcriptome %**	***B. terrestris* DET**	***B. terrestris* DET %**	***T. angustula* transcriptome %**	***T. angustula* DET**	***T. angustula* DET %**
Present only in all *Bombus* [**bumblebees**]	77	0.46%	0.63%	13	1.35%	n.a.	n.a.	n.a.
Present only in both *B. terrestris*, ignoring the absents in the genome [**bterrestris (G)**]	1284	7.73%	10.58%	165	17.15%	n.a.	n.a.	n.a.
Present only in both *B. terrestris* [**specie-specific**]	108	0.65%	0.89%	12	1.25%	n.a.	n.a.	n.a.
Present only in the Stingless bees, ignoring the absents in *F. varia* [**stingless bees (F)**]	97	0.58%	n.a.	n.a.	n.a.	0.77%	4	2.55%
Present only on the Stingless bees [**stingless bees**]	10	0.06%	n.a.	n.a.	n.a.	0.08%	1	0.64%
Present only in *T. angustula* [**specie-specific**]	1944	11.71%	n.a.	n.a.	n.a.	15.36%	40	25.48%
Present only in all corbiculates [**corbiculates**]	81	0.49%	0.67%	4	0.42%	0.64%	0	0.00%
Present only in all social corbiculates [**social corbiculates**]	3	0.02%	0.02%	0	0.00%	0.02%	0	0.00%
present in all species [**apinae**]	6326	38.10%	52.11%	449	46.67%	49.97%	69	43.95%

Table SV Complete list of genes differentially expressed common between *A. mellifera* and *B. terrestris*

A. mellifera* head and *B. terrestris	**common high in nurses?**	**common high in foragers?**	**other body parts DE**
[1] "a disintegrin and metalloproteinase with thrombospondin motifs 7-like"		yes	
[2] "atrial natriuretic peptide receptor 1"			
[3] "basement membrane-specific heparan sulfate proteoglycan core protein"			abdomen, thorax
[4] "cadherin-89d"			abdomen, thorax
[5] "choline/ethanolamine kinase"			abdomen, thorax
[6] "cytochrome c"	yes		
[7] "cytochrome p450 6a2"		yes	thorax
[8] "dentin sialophosphoprotein-like"			abdomen
[9] "ef-hand domain-containing family member c2-like"			
[10] "elongation of very long chain fatty acids protein 6"			abdomen, thorax
[11] "excitatory amino acid transporter"		yes	abdomen, thorax
[12] "facilitated trehalose transporter tret1"		yes	abdomen, thorax
[13] "fatty acyl-coa reductase 1"		yes	abdomen
[14] "golgin-84"			
[15] "heparan-alpha-glucosaminide n-acetyltransferase-like"	yes		
[16] "histone h3"			abdomen
[17] "hmg box-containing protein 4"			thorax
[18] "innexin"	yes		thorax
[19] "lachesin"	yes		thorax
[20] "major royal jelly protein 1"			abdomen, thorax
[21] "maternal protein exuperantia"		yes	thorax
[22] "molybdenum cofactor sulfurase 1"		yes	thorax
[23] "mucin-19-like"			abdomen
[24] "mucin-2-like"			abdomen
[25] "myosin-2 heavy chain"		yes	
[26] "nicotinate phosphoribosyltransferase"		yes	abdomen
[27] "nuclear hormone receptor ftz-f1"			thorax
[28] "protein 5nuc-like"		yes	abdomen
[29] "protein diaphanous"		yes	
[30] "protein inscuteable homolog"			abdomen, thorax
[31] "protein lunapark-b"	yes		thorax
[32] "protein quiver"			thorax
[33] "protein scarlet"	yes		
[34] "proton-coupled amino acid transporter 1"		yes	abdomen, thorax
[35] "putative inorganic phosphate cotransporter"			abdomen, thorax

[36] "rho guanine nucleotide exchange factor 12"			
[37] "scavenger receptor class b member 1"			abdomen, thorax
[38] "sialin"		yes	abdomen, thorax
[39] "sodium-independent sulfate anion transporter-like"		yes	abdomen, thorax
[40] "sodium/hydrogen exchanger"		yes	thorax
[41] "tyrosine-protein kinase btk29a"	yes		abdomen, thorax
[42] "udp-n-acetylglucosamine--peptide n-acetylglucosaminyltransferase 110 kda subunit"			abdomen
A. mellifera thorax* and *B. terrestris			
[1] "14-3-3 protein zeta"		yes	
[2] "activating molecule in becn1-regulated autophagy protein 1-like"	yes		
[3] "adenosine deaminase cecr1-like"	yes		abdomen
[4] "aftiphilin"		yes	
[5] "alpha-tubulin n-acetyltransferase"			abdomen
[6] "aminopeptidase n"			
[7] "apoptosis-inducing factor 3"			
[8] "basement membrane-specific heparan sulfate proteoglycan core protein"			abdomen, head
[9] "cadherin-89d"			abdomen, head
[10] "calcium-binding mitochondrial carrier protein aralar1"		yes	
[11] "cathepsin l1"	yes		
[12] "choline/ethanolamine kinase"			abdomen, head
[13] "chromatin assembly factor 1 subunit b"	yes		
[14] "coronin"			
[15] "cytochrome p450 6a2"		yes	head
[16] "dep domain-containing protein 5"		yes	
[17] "dna ligase 1"	yes		
[18] "dna polymerase theta"			
[19] "dna polymerase zeta catalytic subunit"			
[20] "dopamine n-acetyltransferase"			abdomen
[21] "down syndrome cell adhesion molecule-like protein dscam2"			abdomen
[22] "elongation of very long chain fatty acids protein"			abdomen, head
[23] "epidermal growth factor receptor"			abdomen
[24] "esterase b1-like"			abdomen
[25] "excitatory amino acid transporter"		yes	abdomen, head
[26] "exosome complex component rrp45-like"	yes		
[27] "facilitated trehalose transporter tret1"		yes	abdomen, head
[28] "facilitated trehalose transporter tret1-2 homolog"		yes	

[29] "fibrillin-2"		yes	abdomen
[30] "hemocytin"		yes	
[31] "hmg box-containing protein 4"			head
[32] "innexin"	yes		head
[33] "integral membrane protein gpr155"		yes	
[34] "integrator complex subunit 8"		yes	
[35] "lachesin"			head
[36] "major royal jelly protein 1"	yes		abdomen, head
[37] "maternal protein exuperantia"		yes	head
[38] "microtubule-associated protein futsch"		yes	abdomen
[39] "mitotic spindle assembly checkpoint protein mad2a"	yes		
[40] "mlx-interacting protein"		yes	
[41] "molybdenum cofactor sulfurase 1"		yes	head
[42] "niemann-pick c1 protein-like"		yes	
[43] "nose resistant to fluoxetine protein 6-like"		yes	abdomen
[44] "nuclear hormone receptor ftz-f1"			head
[45] "palmitoyltransferase"			abdomen
[46] "peroxidase"		yes	abdomen
[47] "phosphatidylinositol 4-phosphate 3-kinase c2 domain-containing subunit beta"		yes	abdomen
[48] "phosphodiesterase"			
[49] "phospholipid-transporting atpase"		yes	abdomen
[50] "piezo-type mechanosensitive ion channel component"		yes	
[51] "proteasome subunit alpha type"			
[52] "protein inscuteable homolog"			abdomen, head
[53] "protein lunapark-b"	yes		head
[54] "protein quiver"		yes	head
[55] "protein takeout-like"	yes		abdomen
[56] "protein tilb homolog"		yes	abdomen
[57] "protein yellow"	yes		abdomen
[58] "proton-coupled amino acid transporter 1"		yes	abdomen, head
[59] "proton-coupled amino acid transporter 4"		yes	
[60] "putative fatty acyl-coa reductase cg5065"			abdomen
[61] "putative inorganic phosphate cotransporter"		yes	abdomen, head
[62] "putative phospholipase b-like lamina ancestor"		yes	
[63] "rna-binding protein 1"			
[64] "rna-binding protein fusilli"		yes	abdomen
[65] "scavenger receptor class b member 1"		yes	abdomen, head
[66] "serine/threonine-protein kinase tricorner"		yes	
[67] "sialin"		yes	abdomen, head

[68] "slit homolog 1 protein-like"		yes	
[69] "sodium-independent sulfate anion transporter-like"		yes	abdomen, head
[70] "sodium/hydrogen exchanger"		yes	head
[71] "solute carrier organic anion transporter family member 4a1"		yes	
[72] "stress response protein nst1"			
[73] "targeting protein for xklp2-like"			
[74] "tectonin beta-propeller repeat-containing protein"		yes	
[75] "telomerase reverse transcriptase"			abdomen
[76] "thioredoxin domain-containing protein"			
[77] "thymidylate synthase"	yes		
[78] "titin"		yes	
[79] "tld domain-containing protein 2"		yes	
[80] "transcription factor spt20 homolog"			
[81] "tyrosine-protein kinase btk29a"	yes		abdomen, head
[82] "zinc finger hit domain-containing protein 3"	yes		
A. mellifera abdomen* and *B. terrestris			
[1] "40s ribosomal protein s6"	yes		
[2] "adenosine deaminase cecr1-like"	yes		thorax
[3] "alpha-tubulin n-acetyltransferase"	yes		thorax
[4] "ankyrin repeat domain-containing protein 7-like"			
[5] "atp-dependent (s)-nad(p)h-hydrate dehydratase"			
[6] "basement membrane-specific heparan sulfate proteoglycan core protein"			head, thorax
[7] "cadherin-89d"			head, thorax
[8] "chitobiosyldiphosphodolichol beta-mannosyltransferase"		yes	
[9] "choline/ethanolamine kinase"			head, thorax
[10] "clathrin heavy chain"			
[11] "cryptochrome 2"			
[12] "denn domain-containing protein 5b"	yes		
[13] "dentin sialophosphoprotein-like"			head
[14] "dopamine n-acetyltransferase"			thorax
[15] "down syndrome cell adhesion molecule"			thorax
[16] "elongation of very long chain fatty acids protein aael008004"	yes		head, thorax
[17] "embryonic polarity protein dorsal"	yes		
[18] "epidermal growth factor receptor"			thorax
[19] "esterase b1-like"			thorax
[20] "excitatory amino acid transporter 1"		yes	head, thorax

[21] "facilitated trehalose transporter tret1"		yes	head, thorax
[22] "fatty acyl-coa reductase 1"		yes	head
[23] "fibrillin-2-like"			thorax
[24] "fibroin heavy chain"		yes	
[25] "flavin-containing monooxygenase"		yes	
[26] "glycogen synthase kinase-3 beta"	yes		
[27] "glycolipid transfer protein"			
[28] "golgin subfamily a member 6-like protein 22"	yes	yes	
[29] "histone h3"			head
[30] "lysozyme"		yes	
[31] "major royal jelly protein 1"	yes		head, thorax
[32] "microtubule-associated protein futsch"			thorax
[33] "mitogen-activated protein kinase"			
[34] "mucin-19-like"			head
[35] "mucin-2-like"			head
[36] "muscle m-line assembly protein unc-89"			
[37] "nicotinate phosphoribosyltransferase"		yes	head
[38] "nose resistant to fluoxetine protein 6-like"			thorax
[39] "nuclear receptor subfamily 1 group d member 2"			
[40] "nucleolar protein 58"	yes		
[41] "palmitoyltransferase"		yes	thorax
[42] "peptide transporter family 1"			
[43] "peroxidase"		yes	thorax
[44] "phosphatidylinositol 4-phosphate 3-kinase c2 domain-containing subunit beta"		yes	thorax
[45] "phospholipid-transporting atpase"			thorax
[46] "pre-mrna-processing-splicing factor 8"			
[47] "protein 5nuc-like"		yes	head
[48] "protein claret segregational"	yes		
[49] "protein downstream neighbor of son homolog"	yes		
[50] "protein inscuteable homolog"			head, thorax
[51] "protein ndrg3"			
[52] "protein takeout-like"	yes		thorax
[53] "protein tilb homolog"			thorax
[54] "protein yellow"	yes		thorax
[55] "proton-coupled amino acid transporter 1"		yes	head, thorax
[56] "putative fatty acyl-coa reductase cg5065"			thorax
[57] "putative inorganic phosphate cotransporter"			head, thorax
[58] "putative mediator of rna polymerase ii transcription subunit 26"			
[59] "regulator of microtubule dynamics protein 1-like"		yes	

[60] "reticulon-1"	yes		
[61] "rna-binding protein fusilli"			thorax
[62] "s-phase kinase-associated protein 2"	yes		
[63] "scavenger receptor class b member 1"			head, thorax
[64] "serine/threonine-protein kinase mark2"			
[65] "sialin"		yes	head, thorax
[66] "sodium-independent sulfate anion transporter-like"		yes	head, thorax
[67] "telomerase reverse transcriptase"			thorax
[68] "transmembrane protein 8b"		yes	
[69] "tyrosine-protein kinase btk29a"	yes		head, thorax
[70] "u3 small nucleolar ribonucleoprotein protein imp3"			
[71] "udp-glucose 6-dehydrogenase"		yes	
[72] "udp-n-acetylglucosamine--peptide n-acetylglucosaminyltransferase 110 kda subunit"			head
[73] "unconventional myosin-xv"			
[74] "vascular endothelial growth factor receptor 1"			

Table SVI Complete list of genes differentially expressed common between *A. mellifera* and *T. angustula*

A. mellifera* head and *T. angustula	**common high in nurses?**	**common high in foragers?**	**other body parts DE**
[1] "3-hydroxyacyl-coa dehydrogenase type-2"	yes		abdomen, thorax
[2] "alpha-glucosidase"		yes	thorax
[3] "alpha-mannosidase"	yes		thorax
[4] "basement membrane-specific heparan sulfate proteoglycan core protein"	yes		abdomen, thorax
[5] "cytochrome b"			abdomen, thorax
[6] "cytochrome c"	yes		
[7] "cytochrome p450"		yes	abdomen, thorax
[8] "cytochrome p450 4c1"			
[9] "glucosylceramidase"			abdomen, thorax
[10] "high affinity copper uptake protein 1"			thorax
[11] "histone h2b"			thorax
[12] "histone h3"			abdomen
[13] "hyaluronan mediated motility receptor"			thorax
[14] "lysosomal alpha-mannosidase-like"			
[15] "mitochondrial cardiolipin hydrolase"			
[16] "mucin-2-like"	yes		abdomen
[17] "peptidoglycan-recognition protein"			abdomen, thorax
[18] "prisilkin-39"	yes		abdomen, thorax

[19] "pupal cuticle protein c1b-like"	yes		
[20] "venom acid phosphatase acph-1-like"		yes	thorax
[21] "zinc transporter 1"			
A. mellifera thorax* and T. *angustula			
[1] "3-hydroxyacyl-coa dehydrogenase type-2"	yes		abdomen, head
[2] "alpha-glucosidase"		yes	head
[3] "alpha-mannosidase"	yes		head
[4] "basement membrane-specific heparan sulfate proteoglycan core protein"	yes		abdomen, head
[5] "caspase-1"	yes		
[6] "cathepsin l"	yes		
[7] "cytochrome b"	yes		abdomen, head
[8] "cytochrome p450"		yes	abdomen, head
[9] "glucosylceramidase"			abdomen, head
[10] "high affinity copper uptake protein 1"	yes		head
[11] "histone h2b"	yes		head
[12] "hyaluronan mediated motility receptor"			head
[13] "hydroxysteroid dehydrogenase-like protein 2"		yes	
[14] "lipase 3"	yes		abdomen
[15] "metalloendopeptidase"	yes		
[16] "ornithine decarboxylase"		yes	abdomen
[17] "peptidoglycan-recognition protein"			abdomen, head
[18] "phospholipase a1"	yes		
[19] "prisilkin-39"	yes		abdomen, head
[20] "protein fam13a"		yes	
[21] "putative fatty acyl-coa reductase cg5065"		yes	abdomen
[22] "targeting protein for xklp2"			
[23] "trypsin-1"			abdomen
[24] "venom acid phosphatase acph-1-like"		yes	head
A. mellifera abdomen* and *T. angustula			
[1] "3-hydroxyacyl-coa dehydrogenase type-2"	yes		head, thorax
[2] "anion exchange protein"		yes	
[3] "basement membrane-specific heparan sulfate proteoglycan core protein"	yes		head, thorax
[4] "carboxypeptidase b-like"	yes		
[5] "chymotrypsin-1"			
[6] "cytochrome b"	yes		head, thorax
[7] "cytochrome p450"		yes	head, thorax
[8] "glucosylceramidase"			head, thorax
[9] "glucosylceramidase-like"	yes		

[10] "helicase domino"			
[11] "histone h3"			head
[12] "lipase 3-like"			thorax
[13] "mucin-2-like"	yes		head
[14] "ornithine decarboxylase"		yes	thorax
[15] "pdf receptor"			
[16] "peptidoglycan-recognition protein"			head, thorax
[17] "prisilkin-39"	yes		head, thorax
[18] "protein g12"	yes		
[19] "putative fatty acyl-coa reductase cg5065"		yes	thorax
[20] "trypsin-1"			thorax
[21] "vanin-like protein 1"			

Table SVII Complete list of genes differentially expressed common between *B. terrestris* and *T. angustula*

B. terrestris* and *T. angustula	**common high in nurses?**	**common high in foragers?**
[1] "at-rich interactive domain-containing protein 2"		yes
[2] "basement membrane-specific heparan sulfate proteoglycan core protein isoform x1"		
[3] "cathepsin l"	yes	
[4] "chymotrypsin-2-like"	yes	
[5] "cytochrome c oxidase subunit 1"	yes	
[6] "cytochrome p450"		yes
[7] "histone h3"	yes	
[8] "mucin-2-like"		
[9] "putative fatty acyl-coa reductase cg5065"		
[10] "retrovirus-related pol polyprotein from transposon tnt 1-94"	yes	
[11] "sec23-interacting protein"		
[12] "targeting protein for xklp2"	yes	
[13] "transposable element tc1 transposase"	yes	
[14] "tubulin glycylase 3a"		
[15] "urea transporter 1-like"		

Supplementary Figures

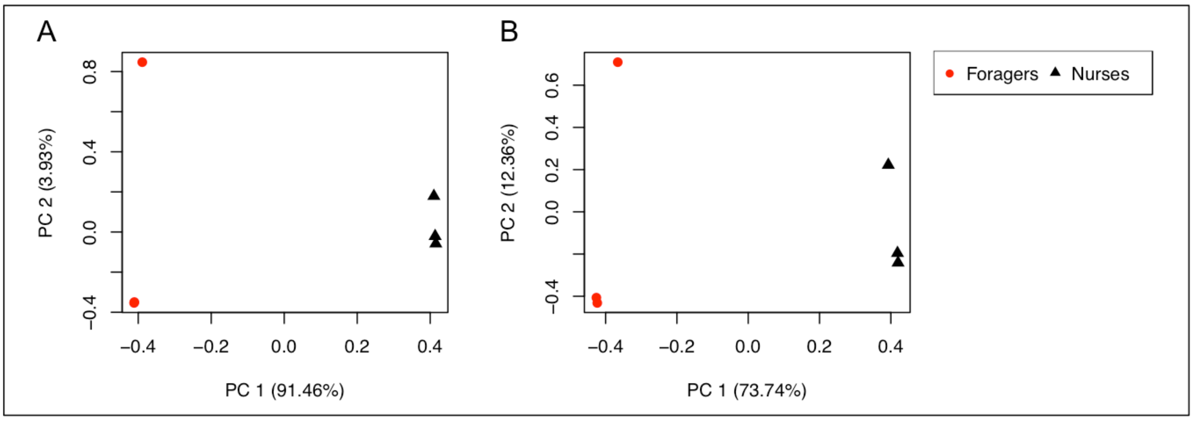

Figure S1 Principal Component Analyses using superTranscripts normalized counts by DESeq2. The two principal components involved in sample differences are shown. **A-** *B. terrestris* data. **B-** *T. angustula* data.

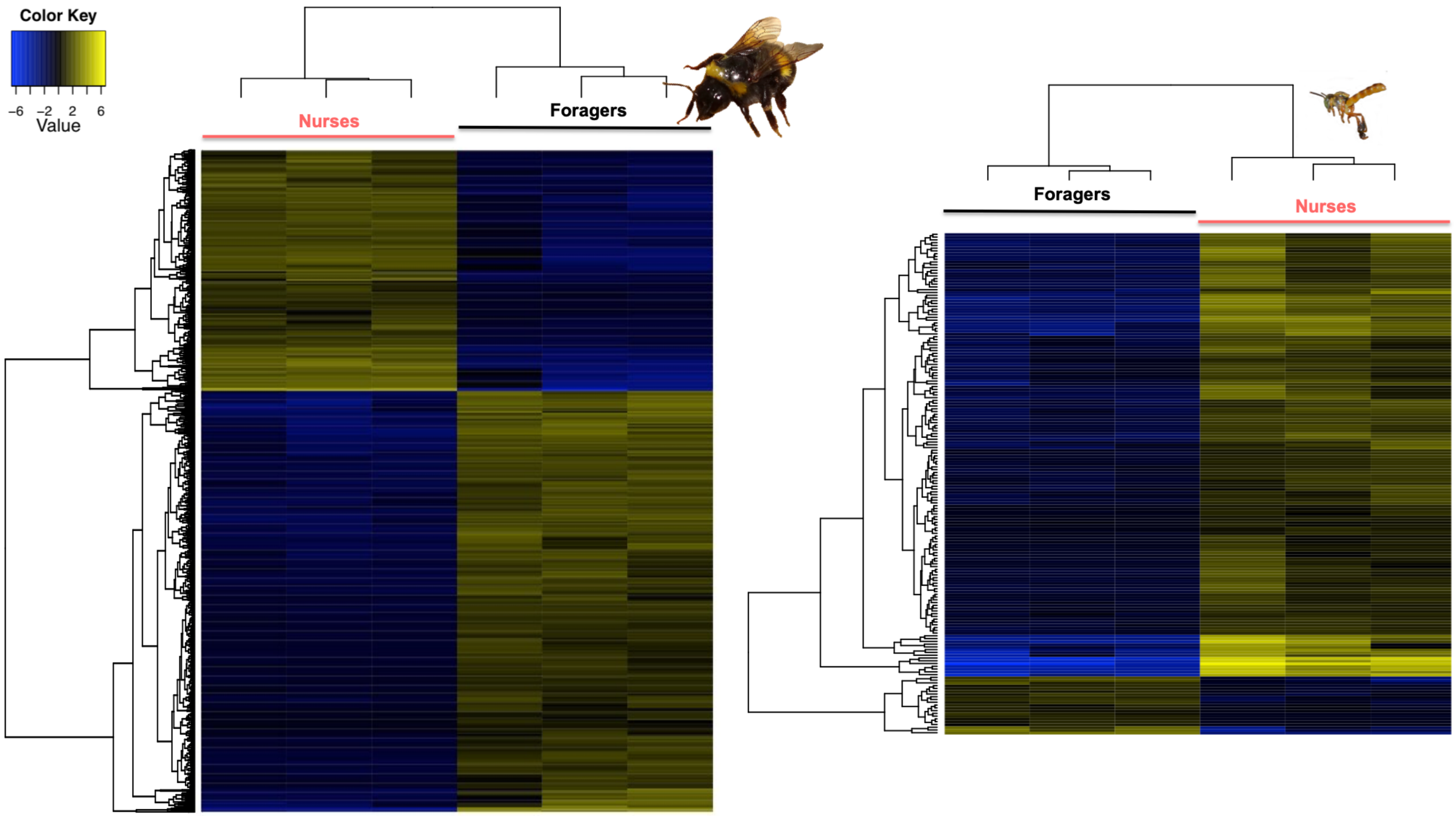

Figure S2 Heatmap of the differentially expressed superTranscripts between foragers and nurses of *B. terrestris* (left; 1,203 superTranscripts) and *T. angustula* (right; 241 superTranscripts). Each row represents one superTranscript while columns are distinct sample replicates. Expression scale is on log2 fold change (logFC) and minimum differential expression difference between groups is of 2*logFC, i.e. approximately four-fold difference between groups. *B. terrestris* and *T. angustula* photo sizes are not scaled. *T. angustula* by L. Costa – image reproduced with permission from the original author.

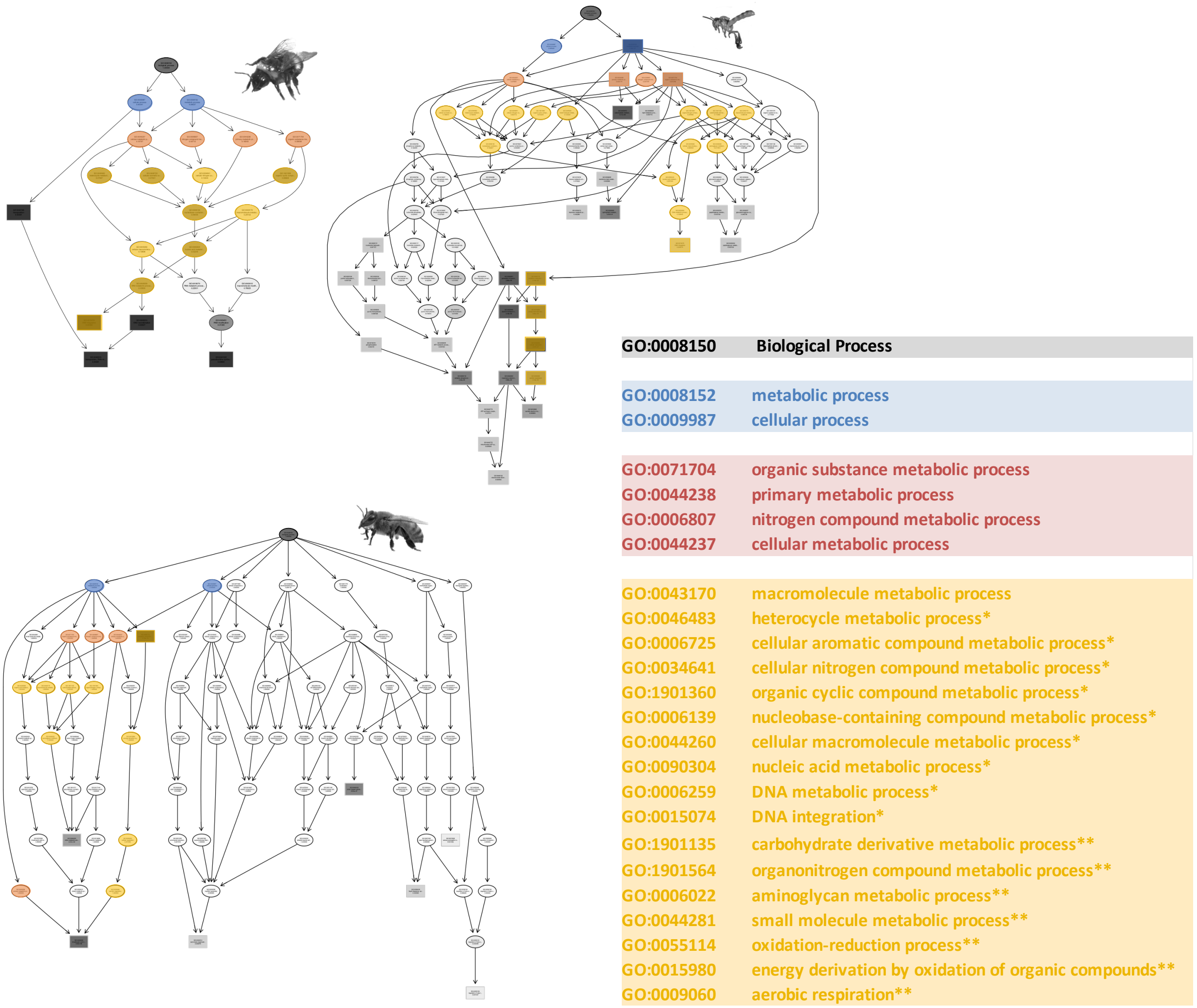

Figure S3 Summarized overlap between subgraphs induced by the BP terms found enriched in the differentially expressed genes of all species. Colours highlight common BP terms in species subgraphs: black – common in the first hierarchical level; blue – common in the second hierarchical level; red – common in the third hierarchical level; yellow – common in all other levels. Squares represent significantly enriched terms (p < 0.01) see Figures S4, S5 and S6for details. The subgraph of the top 8 GO terms enriched in *A. mellifera* is shown. * indicate terms common only to *B. terrestris* and *T. angustula*, ** terms common to *A. mellifera* and *T. angustula*. *A. mellifera* by A. Wide, *T. angustula* by L. Costa – images reproduced with permission from the original authors.

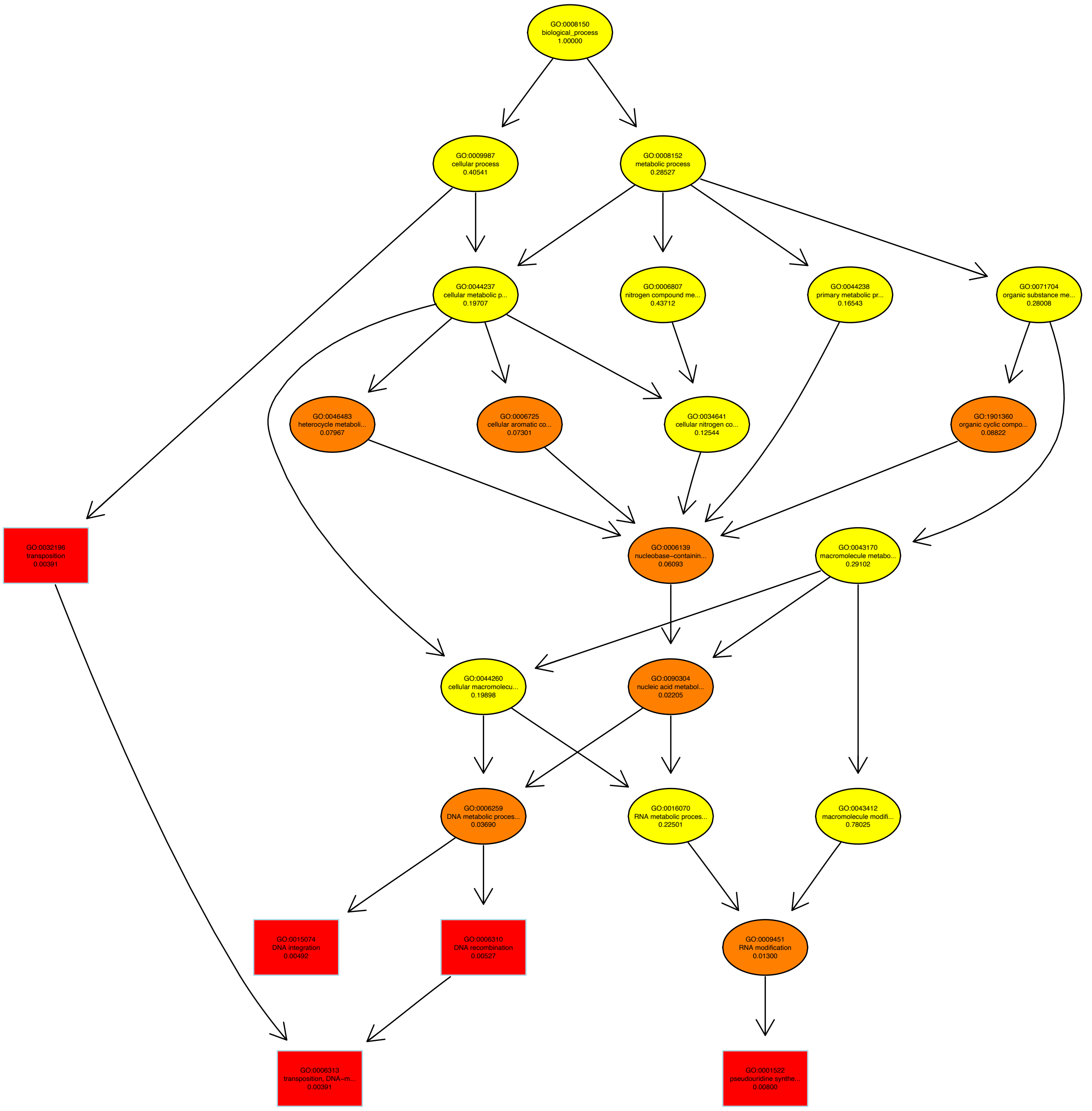

Figure S4 Subgraph induced by the enriched GO terms of biological processes found among the differentially expressed superTranscripts of *B. terrestris* (p< 0.01) when compared to the entire transcriptome. GO terms identified by the classic algorithm for scoring GO terms for enrichment. Box colours represent the relative significance, ranging from dark red (most significant) to light yellow (least significant). Black arrows indicate *is-a* (i.e., is a subtype of) relationships.

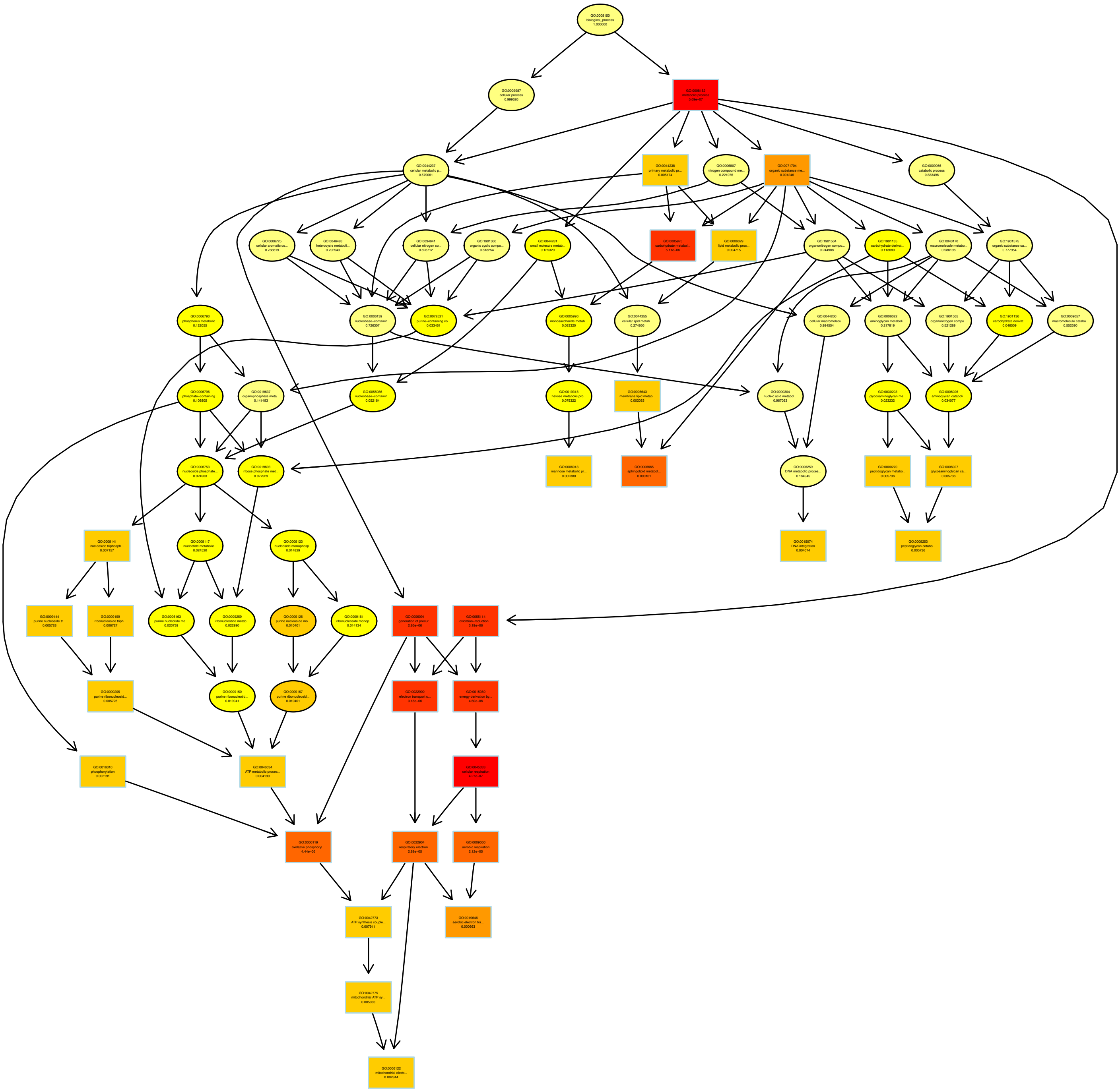

Figure S5 Subgraph induced by the enriched GO terms of biological processes found among the differentially expressed superTranscripts of *T. angustula* ($p< 0.01$) when compared to the entire transcriptome. GO terms identified by the classic algorithm for scoring GO terms for enrichment. Box colours represent the relative significance, ranging from dark red (most significant) to light yellow (least significant). Black arrows indicate *is-a* (i.e., is a subtype of) relationships.

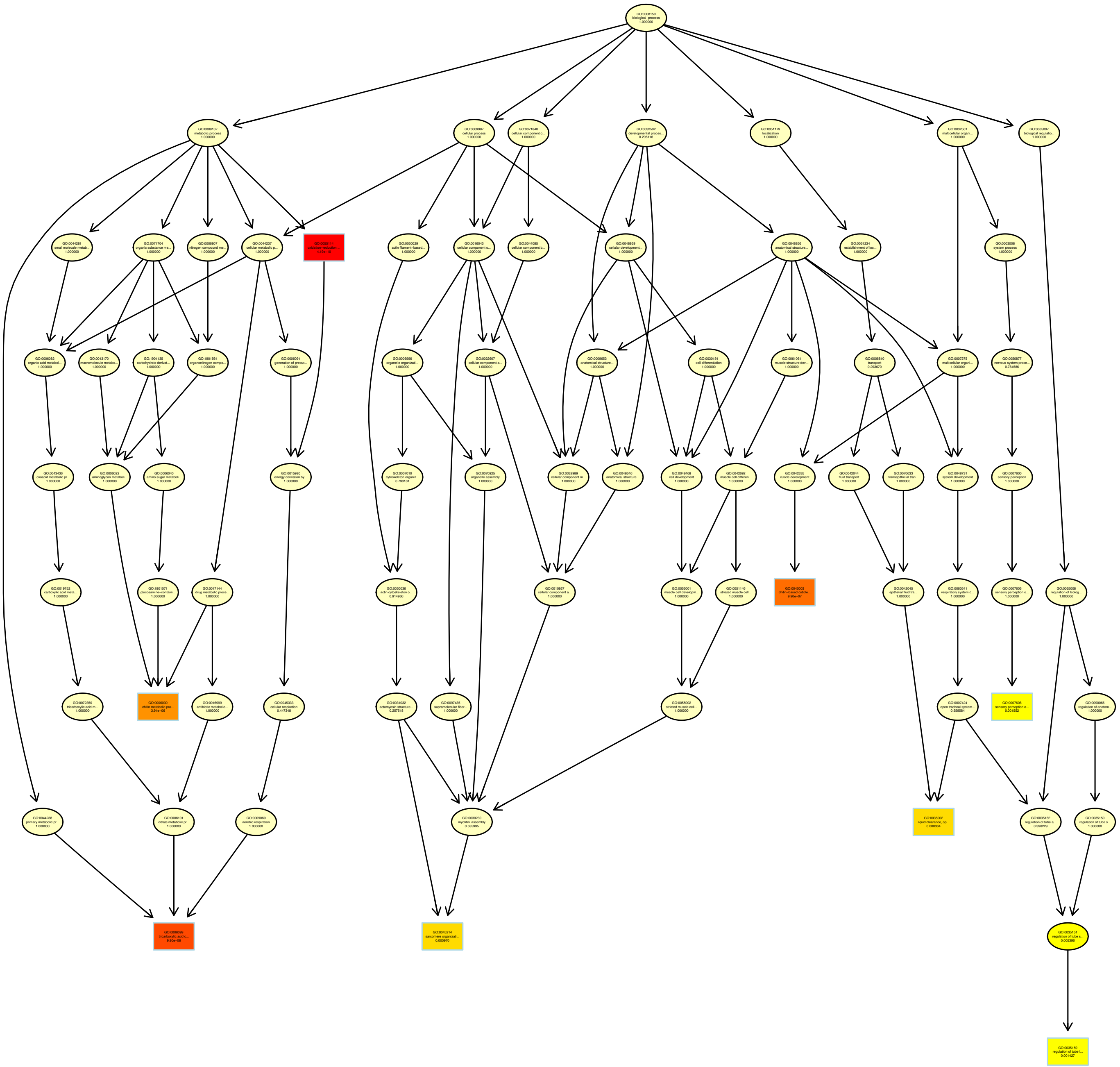

Figure S6 Subgraph induced by the top eight enriched GO terms of biological processes found among the differentially expressed genes in the head of *A. mellifera* ($p< 0.01$) when compared to the expressed transcriptome (as defined by [1]). Top 8 GO terms identified by the weighted01 algorithm for scoring GO terms for enrichment. Box colours represent the relative significance, ranging from dark red (most significant) to light yellow (least significant). Black arrows indicate *is-a* (i.e., is a subtype of) relationships.
The subgraph induced by all enriched GO terms is available at https://github.com/nat2bee/Foragers_vs_Nurses.

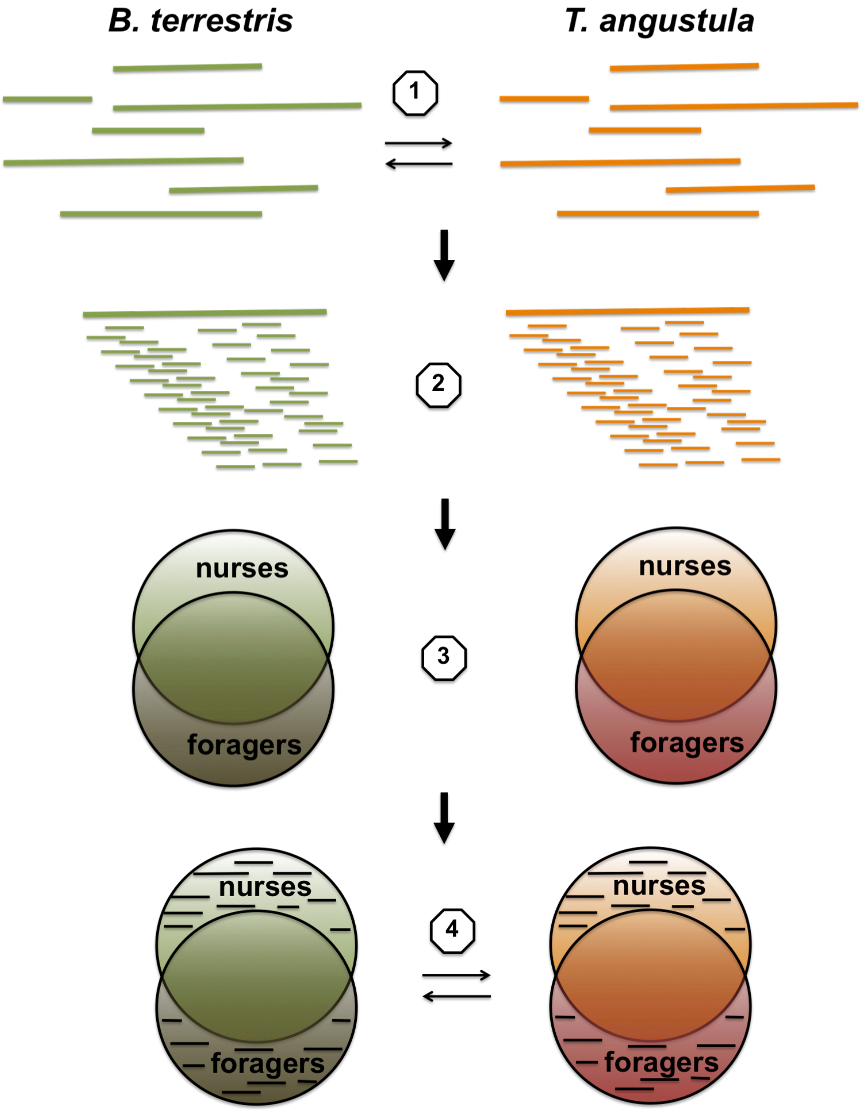

Figure S7. Workflow of analysis comparing expression of nurses and foragers of *T. angustula* and *B. terrestris*. **1** – Transcriptome assembly. **2** - Realignment of the cleaned reads to the assembled superTranscripts for expression count. **3** - Differential expression analyses between the distinct worker subcastes within species. **4** - Comparison of the results between species.

1. Warner, M. R., Qiu, L., Holmes, M. J., Mikheyev, A. S. & Linksvayer, T. A. Convergent eusocial evolution is based on a shared reproductive groundplan plus lineage-specific plastic genes. *Nat. Commun.* **10**, 1–11 (2019).
